# Lysine-specific demethylase 1 regulates hematopoietic stem cell expansion and myeloid cell differentiation

**DOI:** 10.1038/s41419-025-07951-z

**Published:** 2025-08-15

**Authors:** Hans Felix Staehle, Christoph Koellerer, Anne Marie Staehle, Jana Schulze, Philipp Eble, Anja Müller, Franziska Zell, Judith M. Müller, Florian Perner, Aya Attia, Jan-Philipp Mallm, Olga Pozdnyakova, Karsten Rippe, Benedikt Brors, Lars Feuerbach, Charles D. Imbusch, Eric Metzger, Roland Schüle, Heike L. Pahl, Jonas S. Jutzi

**Affiliations:** 1 Division of Molecular Hematology, Department of Medicine I, Medical Center – University of Freiburg, Faculty of Medicine, University of Freiburg, Freiburg, Baden-Württemberg Germany; 2 Faculty of Biology, University of Freiburg, Freiburg, Germany; 3 Department of Urology, University Medical Center Freiburg, Faculty of Medicine, University of Freiburg, Freiburg, Baden-Württemberg Germany; 4 Hematology, Hemostasis, Oncology, and Stem Cell Transplantation, Hannover Medical School, Hannover, Germany; 5 German Cancer Research Center (DKFZ), Single Cell Open Lab, Heidelberg, Baden-Württemberg Germany; 6 Division of Hematopathology, Department of Pathology and Laboratory Medicine, Hospital of the University of Pennsylvania, Pennsylvania, PA USA; 7 German Cancer Research Center (DKFZ), Division of Chromatin Networks, Heidelberg, Germany; 8 Center for Quantitative Analysis of Molecular and Cellular Biosystems (BioQuant), Heidelberg University, Heidelberg, Germany; 9 German Cancer Research Center (DKFZ), Division of Applied Bioinformatics, Heidelberg, Baden-Württemberg Germany; 10 National Center for Tumor Diseases (NCT), Heidelberg, Baden-Württemberg Germany; 11 German Cancer Consortium (DKTK), Core Center Heidelberg, Heidelberg, Germany; 12 Medical Faculty and Faculty of Biosciences, Heidelberg University, Heidelberg, Germany; 13 University Medical Center Mainz, Institute of Immunology, Mainz, Rheinland-Pfalz Germany; 14 University Medical Center Mainz, Research Center for Immunotherapy, Mainz, Rheinland-Pfalz Germany

**Keywords:** Epigenetics, Haematopoietic stem cells, Transcriptomics, Myelopoiesis

## Abstract

The lysine-specific demethylase 1 (LSD1) regulates hematopoietic stem cell differentiation and has been identified as a therapeutic target in hematological disorders. LSD1 demethylates mono and dimethylated histones 3 at lysine 4 and 9. In addition, it acts as a scaffold for the formation of chromatin-modifying complexes that regulates the transcription of myeloid-lineage-specific genes in complex with GFI1, a transcriptional repressor. While both enzymatic and non-enzymatic functions of LSD1 have been well defined, the relative importance of these two functions in hematopoiesis remains incompletely understood. Here, we investigated the contribution of enzymatic and non-enzymatic functions of LSD1 to myelopoiesis. We show that myeloid differentiation is independent of the enzymatic functions of LSD1 but requires the non-enzymatic, scaffolding function, which directs GFI1 binding to target sequences. In the absence of the LSD1 protein, GFI1 DNA binding is diminished, and myeloid cell differentiation arrests at an immature, myelomonocytic-like cell stage, which overexpresses *Prtn3*. We provide functional data implicating *Prtn3* as an effector of the stem cell expansion and myeloid maturation block caused by the loss of LSD1.

## Introduction

The *lysine-specific demethylase 1* (LSD1, *a.k.a*. KDM1A) regulates hematopoietic stem cell (HSC) differentiation and has been identified as a therapeutic target in a wide range of hematological disorders [1–8]. LSD1 functions as a histone demethylase, demethylating mono and dimethylated histones 3 at lysine 4 and 9 [9, 10]. In addition, LSD1 acts as a scaffold for the formation of chromatin-modifying complexes. In these complexes, LSD1 regulates transcription by interacting with the myeloid-lineage-specific transcriptional repressor GFI1 [11]. Thus, LSD1 exerts both enzymatic and non-enzymatic functions. However, the relative importance of these two functions for the role of LSD1 in hematopoiesis has not been investigated in detail.

A global loss of the LSD1 protein, resulting in a loss of both the enzymatic and the nonenzymatic function, causes hematopoietic maturation defects, most pronounced in the myeloid lineage [12, 13]. In acute myelogenous leukemia (AML)-derived cell lines, nonenzymatic activities of LSD1 include the recruitment of LSD1-containing complexes to chromatin as well as the interaction between LSD1 and GFI1, an essential transcription factor for myeloid differentiation [5]. However, the mechanisms leading to myeloid maturation arrest upon loss of the LSD1 protein in healthy hematopoiesis are not known.

It has been shown that GFI1 antagonizes the activity of the myeloid transcription factor SPI1 (*a.k.a*. PU.1) by direct physical interaction [14]. Ectopic expression of *Gfi1* in murine hematopoietic stem and progenitor cells (HSPCs) increases granulocyte differentiation at the expense of macrophage differentiation. Vice versa, myeloid cells depleted of GFI1 overexpress SPI1 target genes, for example, *SPI1* itself and *M-CSF* [14]. In *Gfi1* ko mice, this leads to an abnormal population of CD11b^+^ Gr1^+^ cells sharing characteristics of both granulocytes and macrophages [15]. Attenuation of SPI1 activity by GFI1 is thus critical for the downregulation of macrophage gene expression, and this is necessary for physiological granulocyte maturation [14]. However, SPI1 activity is essential for the initial steps of myeloid maturation, as mice lacking *Spi1* do not develop either mature macrophages or granulocytes [16, 17].

SPI1 drives expression of the myeloid-specific genes proteinase 3 (*Prtn3)* [18] and neutrophil elastase (*Elane)* [19]. *Prtn3* and *Elane* play important roles in HSC homeostasis, myeloid lineage commitment, and differentiation. *Prtn3* is highly expressed in both HSCs and hematopoietic stem and progenitor cells (HSPCs) and has been shown to regulate cell survival and engraftment of HSPCs [20]. Moreover, overexpression of *Prtn3* leads to cytokine-independent growth of hematopoietic cells in vitro, but this effect has not been verified in vivo [21]. Mutations in *ELANE* were described in patients with severe congenital neutropenia, which show impaired granulocytic maturation [22]. In monocytic cell lines as well as in primary monocytes, repression of PRTN3- and ELANE-mediated histone H3 proteolytic cleavage promoted macrophage differentiation [23].

Here, we investigated the relative contribution of the enzymatic and the non-enzymatic functions of LSD1 to myelopoiesis. We show that myeloid differentiation is independent of the enzymatic functions of LSD1 but requires the non-enzymatic function, which directs GFI1 binding to target sequences. In absence of the LSD1 protein, GFI1 DNA binding is diminished, and myeloid cell differentiation arrests at an immature, myelomonocytic-like cell stage, which overexpresses *Prtn3*. We provide functional data implicating *Prtn3* as an effector of the stem cell expansion and myeloid cell differentiation block caused by loss of LSD1.

## Results

### *Lsd1* knockout mice accumulate immature myeloid cells in the bone marrow

To elucidate mechanisms causing the hematopoietic phenotype observed following either loss of LSD1 protein expression or loss of LSD1 enzymatic function, we used two complementary mouse models: (i) a conditional *Lsd1* knockout (ko) mouse strain, which displays a complete loss of LSD1 protein expression upon tamoxifen induction (*Lsd1*^tm1Schüle^, here termed *Lsd1*^fl/fl^ ko) [24] and (ii) a conditional *Lsd1* knock-in mouse strain, in which tamoxifen induction causes expression of an altered LSD1 allele, which carries three point mutations in the amino oxidase domain, rendering it enzymatically inactive, but maintaining protein stability (*Lsd1*^K661A, W752A, Y762S^, here termed *Lsd1*^fl/fl^ ei) [25].

In the first set of experiments, we crossed *Lsd1*^fl/fl^ ko and *Lsd1*^fl/fl^ ei mice with Rosa26-Cre-ERT2 mice, which express Cre under the control of an inducible estrogen receptor. In double transgenic mice, Cre-induced recombination via tamoxifen injections resulted in *Lsd1* ko and *Lsd1* ei mice, in which the *Lsd1* locus is altered in all cells of the body. *Lsd1*^fl/fl^ ko and *Lsd1*^fl/fl^ ei mice without Cre were used as controls, henceforth called *Lsd1* ko ctrl and *Lsd1* ei ctrl, respectively. Effective Tamoxifen-induced allelic recombination in vivo was demonstrated in *Lsd1* ei mice (Supplementary Fig. 1). Furthermore, we used ChIP-seq to functionally validate the loss of LSD1 enzymatic activity in *Lsd1* ei mice, demonstrating an extensive change in global prevalence of the H3K4me1 histone mark, which is targeted by LSD1 (Supplementary Fig. 2) [26].

Sprüssel *et al*. have previously reported pancytopenia following shRNA-mediated *Lsd1* knockdown [13]. Similarly, Kerenyi et al*.* showed that *Mx1-Cre* or *Vav-Cre*-driven deletion of *Lsd1* caused pancytopenia [12]. Here, we confirm that complete loss of the LSD1 protein by genetic deletion in all cell types results in severe anemia and a nearly complete loss of leukocytes and platelets (Supplementary Fig. 3A–C).

**Fig. 1:**
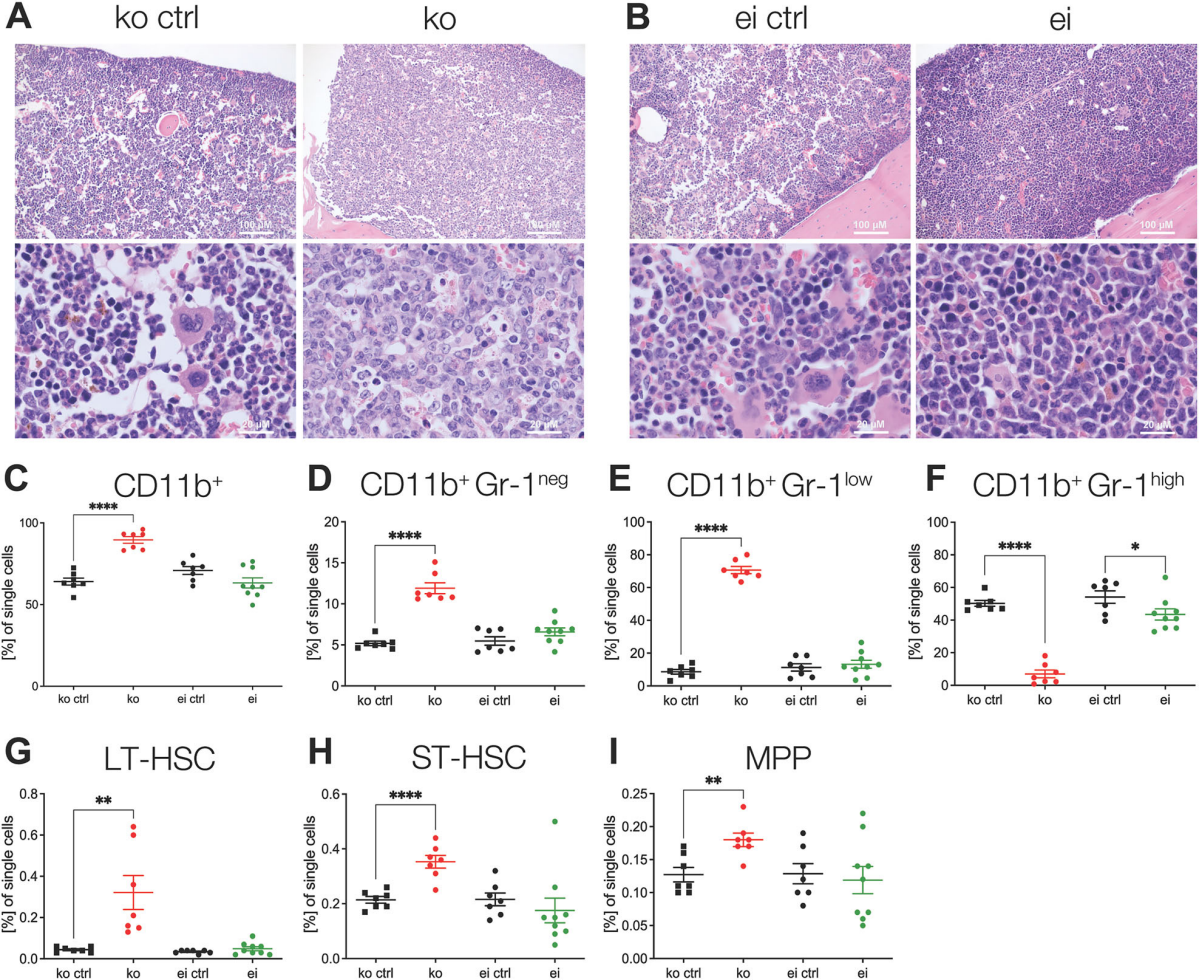
*Lsd1* knockout mice display increased hematopoietic stem and progenitor cells and immature myeloid cells in the bone marrow. **A**–**I** *Lsd1*^*fl/fl*^ mice without Cre served as separate controls in both lines (ko ctrl and ei ctrl). n = 7–9 per genotype. Histopathological BM slides of (**A**) *Lsd1* ko ctrl (left) and *Lsd1* ko (right) as well as (**B**) *Lsd1* ei ctrl (left) and *Lsd1* ei (right) femora. Sections were stained using hematoxylin and eosin. 200x magnification (top) and 1000x magnification (bottom). **C**–**I** Frequency of myeloid cells and hematopoietic stem and progenitor cells in the BM of *Lsd1* ko and *Lsd1* ei mice by flow cytometry analysis. Statistical testing was performed using Student’s *t* tests. *p < 0.05, **p < 0.01, ****p < 0.0001; n = 7–9 per genotype. Only statistically significant comparisons are denoted. (**C**) CD11b^+^ positive cells, **D** CD11b^+^ Gr-1^neg^, **E** CD11b^+^ Gr-1^low^, **F** CD11b^+^ Gr-1^high^, **G** long-term HSCs (LT-HSC), **H** short-term HSCs (ST-HSC), and **I** multipotent progenitors (MPP).

We subsequently examined the effect of *Lsd1* deletion or enzymatic inactivation in the bone marrow (BM). Bone marrow cellularity was similar in *Lsd1* ko, *Lsd1* ei, and their respective littermate controls (Supplementary Fig. 3D). However, in histological and cytomorphological analyses, *Lsd1* ko ctrl, *Lsd1* ei ctrl, and *Lsd1* ei BMs were physiologically polymorphic, displaying all three major lineages. In contrast, *Lsd1* ko BM appeared homogeneous, containing almost exclusively immature myeloid forms (Fig. 1A+B and Supplementary Fig. 3E+F). Flow cytometry analyses confirmed a drastic expansion in the proportion of CD11b-positive, myeloid cells in *Lsd1* ko BM, where they represented on average 90% of the total cell count (Fig. 1C). The absolute number of CD11b-positive cells remained unaltered (Supplementary Fig. 3G), underlining the drastic lineage bias induced by loss of the LSD1 protein*. Lsd1* ei mice, in contrast, are similar to control animals and contain around 60% CD11b-positive cells (Fig. 1C).

Within the myeloid compartment, monocytic cells (CD11b^+^, Gr-1^neg^) and early, immature granulocytic and monocytic cells (CD11b^+^, Gr-1^low^) [27, 28] were strikingly expanded in *Lsd1* ko BM, while mature granulocytes (CD11b^+^, Gr-1^high^) were nearly absent (Fig. 1D–F and Supplementary Fig. 3H–J). The latter is in line with previous data showing impaired myeloid maturation following *Lsd1* depletion by RNAi [13]. *Lsd1* ei BM shows a maturation of the myeloid lineage comparable to that seen in control BM, albeit with a slight reduction in the proportion of mature granulocytes, demonstrating that the LSD1 scaffolding function plays an important role in hematopoietic lineage determination and maturation while the enzymatic activity is subsidiary. Flow cytometry analyses revealed an expansion of long- and short-term HSCs (LT-HSCs) as well as multipotent progenitors (ST-HSCs and MPPs) in *Lsd1* ko but not in *Lsd1* ei mice (Fig. 1G–I and Supplementary Fig. 3K–M), demonstrating that LSD1 scaffolding function, not its enzymatic activity, is required for HSC homeostasis.

*Lsd1* deficient mice displayed a significantly shortened survival (Supplementary Fig. 3N). We hypothesize that this is due to the severe hematological phenotype, however, *Lsd1* deletion in all tissues may cause additional morbidity. We therefore used BM transplantation to create mice in which LSD1 activity is altered solely in the hematopoietic system. Effects of changes in *Lsd1* expression or activity that are intrinsic to the hematopoietic stem cell will manifest themselves in BM transplant recipients, while cell-extrinsic effects will not be observed in the wild-type (wt) recipient background.

### Hematopoiesis-specific *Lsd1* knockout mice display shorter survival and increased myeloid cells in the BM

**Fig. 2:**
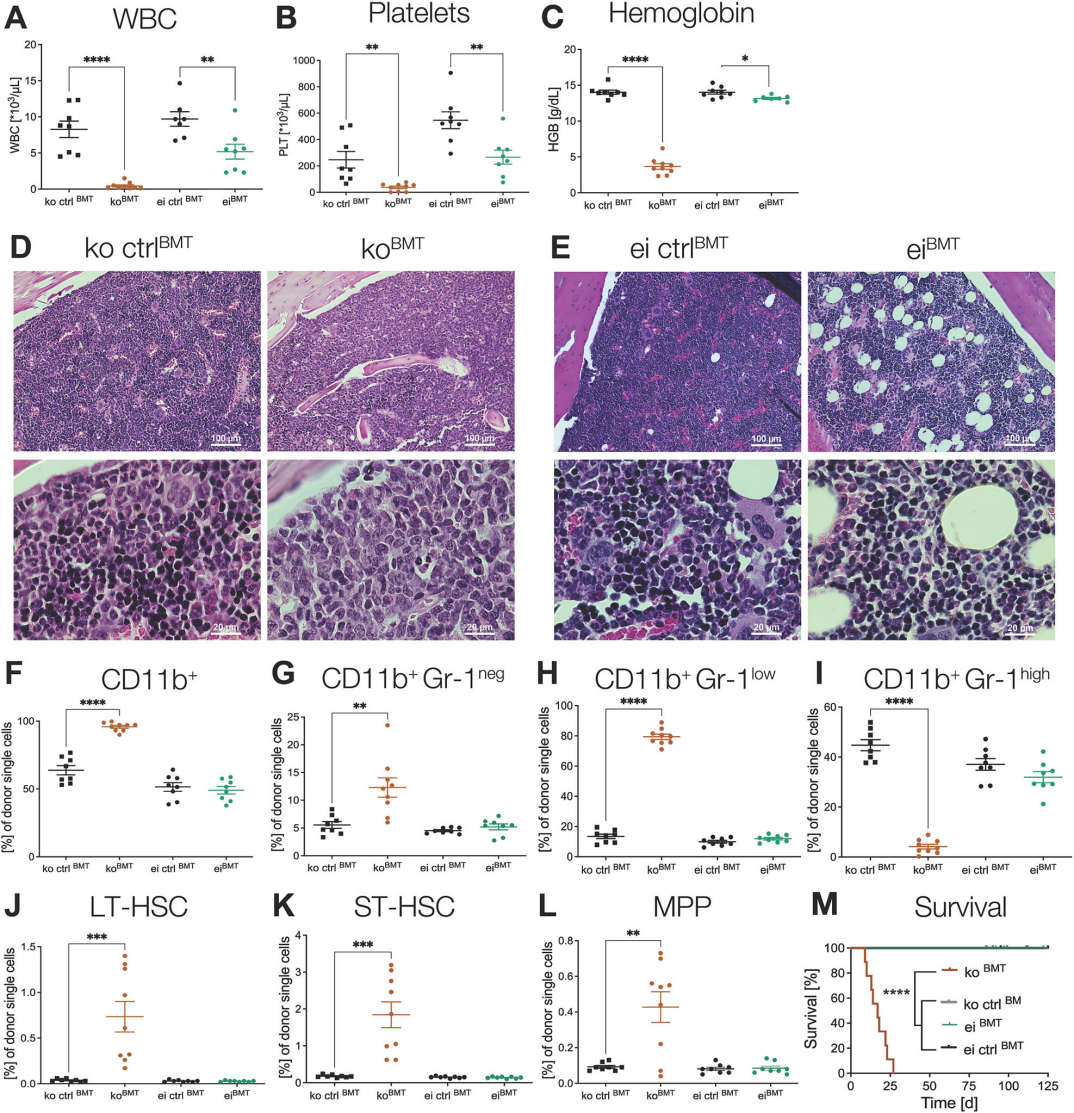
Hematopoiesis-specific *Lsd1* knockout displays shorter survival, pancytopenia, increased hematopoietic stem and progenitor cells, and myeloid cells in the bone marrow. **A**–**M** *Lsd1*^*fl/fl*^ mice without Cre served as separate controls in both mouse lines (ko ctr^BMT^ and ei ctr^BMT^). **A**–**C**, **F**–**L** Statistical analyses of peripheral blood and bone marrow (BM) populations were conducted using Student’s t tests. n = 7–9 per genotype. **M** Survival analysis was performed using Log-Rank (Mantel-Cox) testing. *p < 0.05; **p < 0.01; ***p < 0.001; ****p < 0.0001. n = 7–9 per genotype. Only statistically significant comparisons are denoted. **A** White blood cell counts (WBC), **B** Platelet (PLT) counts, and **C** hemoglobin (HGB) values of *Lsd1* ko^BMT^ and *Lsd1* ei^BMT^ mice. Histopathological BM slides of **D** *Lsd1* ko ctrl^BMT^ (left) and *Lsd1* ko^BMT^ (right), as well as **E** *Lsd1* ei ctrl^BMT^ (left) and *Lsd1* ei^BMT^ (right) femora. 200x magnification (top) and 1000x magnification (down). Sections were stained using hematoxylin and eosin. Frequency of myeloid cells in the BM of *Lsd1* ko^BMT^ and *Lsd1* ei^BMT^ mice by flow cytometry analysis: **F** CD11b^+^ positive cells, **G** CD11b^+^ Gr-1^neg^, **H** CD11b^+^ Gr-1^low^, **I** CD11b^+^ Gr-1^high^. **J**–**L** Hematopoietic stem and progenitor cell (HSPC) frequency of *Lsd1* ko^BMT^ and ei^BMT^ mice in the BM by flow cytometry analysis: **J** long-term HSCs (LT-HSC), **K** short-term HSCs (ST-HSC), and **L** multipotent progenitors (MPP). **M** Kaplan–Meier survival curves of *Lsd1* ko^BMT^ and *Lsd1* ei^BMT^ mice.

To determine whether the hematological phenotype is cell-intrinsic, we thus transplanted BM harvested from primary, total body *Lsd1* ko and *Lsd1* ei mice into lethally irradiated recipients, subsequently termed *Lsd1* ko^BMT^ and *Lsd1* ei^BMT^ mice, adding the appropriate controls, *Lsd1* ko ctrl^BMT^ and *Lsd1* ei ctrl^BMT^. While *Lsd1* ko^BMT^ mice displayed profound pancytopenia, *Lsd1* ei^BMT^ showed only a mild reduction of the three lineages (Fig. 2A–C). Bone marrow cellularity was reduced in both *Lsd1* ko^BMT^ and *Lsd1* ei^BMT^ mice compared to littermate controls (Supplementary Fig. 4A). As in the primary donor animals, BM histopathology and cytomorphology showed an excess of immature myeloid cells in *Lsd1* ko^BMT^ animals, absent in *Lsd1* ei^BMT^ and control animals (Fig. 2D+E and Supplementary Fig. 4B+C). The predominance of CD11b-positive cells in *Lsd1* ko^BMT^ accompanied by the lack of granulocytic maturation is witnessed in the bone marrow transplant (BMT) recipients precisely as described above in the donor, total body ko mice (Fig. 2F–I and Supplementary Fig. 4D–G). Like the total body knockout, *Lsd1* ko^BMT^ mice also exhibited a significant expansion of all HSPC subtypes in the BM, which was not present in *Lsd1* ei^BMT^ mice (Fig. 2J–L and Supplementary Fig. 4H–J). Moreover, while the spleens of *Lsd1* ei^BMT^ and control mice showed physiological histology with delineated white and red pulps, this architecture was destroyed in *Lsd1* ko^BMT^ animals. The latter contains a highly expanded hematogenous red pulp, characteristic of extramedullary hematopoiesis, which results in splenomegaly (Supplementary Fig. 4K–M). Similar to primary *Lsd1* ko mice, *Lsd1* ko^BMT^ mice showed a shortened survival (Fig. 2M). The hematological phenotype is thus cell-intrinsic and transplantable.

Our data demonstrate that isolated *Lsd1* deletion in the hematopoietic system causes a severe phenotype which is rapidly fatal. Of note, survival of *Lsd1* ei^BMT^ was not impaired and comparable to that of control animals (*Lsd1* ko ctrl^BMT^ and *Lsd1* ei ctrl^BMT^, Fig. 2M), suggesting that absence of the LSD1 protein causes a cell-intrinsic defect in HSCs that is not conferred by the mere absence of its enzymatic function. Moreover, our data show that LSD1 scaffolding function is required for both lineage commitment and myeloid maturation, as its loss results in myeloid skewing and in the accumulation of immature myeloid forms. Conversely, we show for the first time that LSD1 enzymatic activity is dispensable for these functions.

### Loss of LSD1 protein expression but not of enzymatic activity leads to distinct clustering in scRNA-seq with upregulated expression of the myeloid protease *Prnt3*

**Fig. 3:**
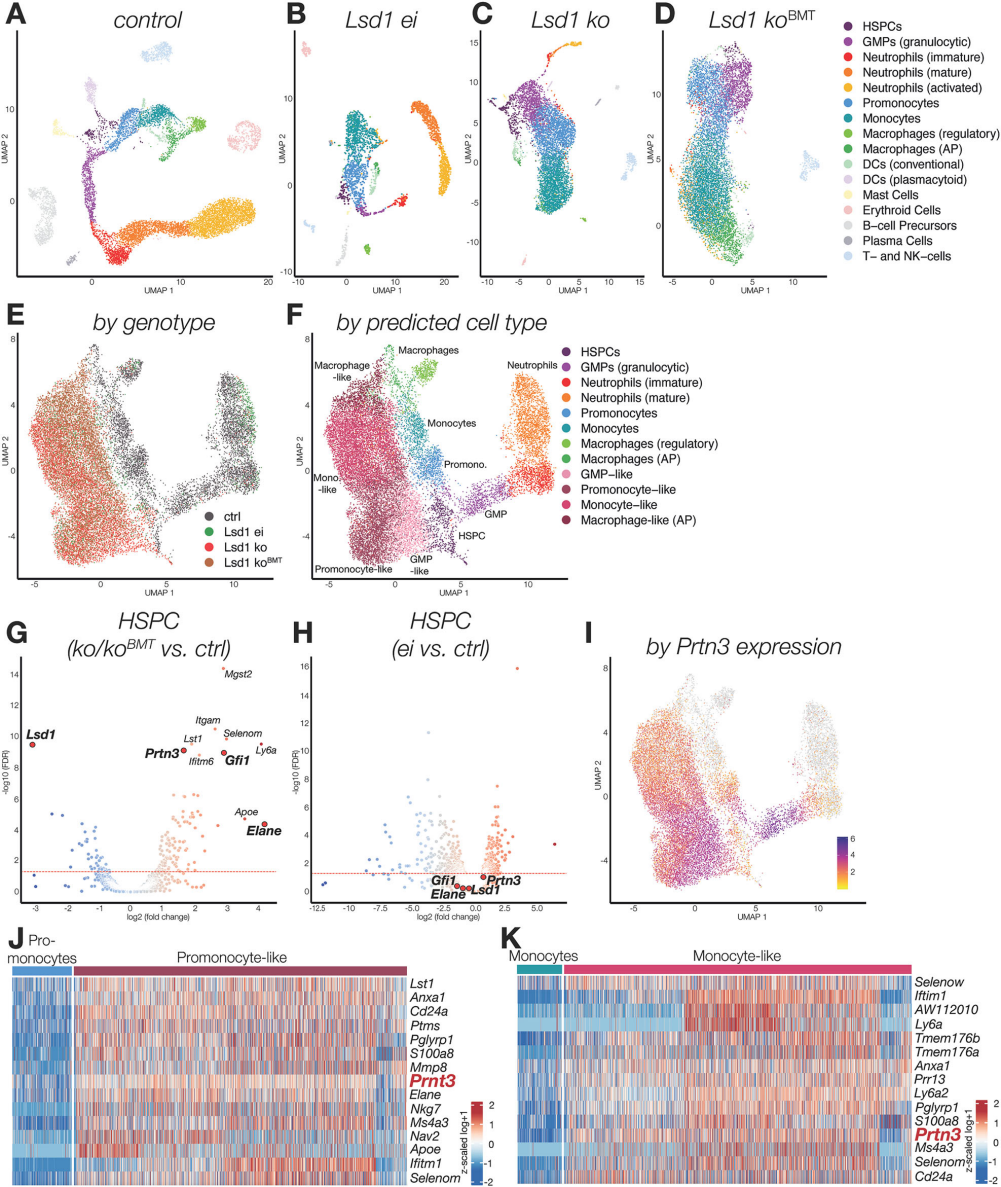
*Lsd1* depletion, but not its enzymatic inactivation, increases *Prtn3* expression resulting in lineage restriction, myeloid expansion and maturation arrest. Single-cell RNA-sequencing (scRNA-seq) of *Lsd1* ei, *Lsd1* ko and *Lsd1* ko^BMT^ BM as well as their littermate controls, n = 28,869 cells. UMAP embeddings colored by cell type (reference or predicted) **A** control (n = 7 mice, *Lsd1* ei ctrl, *Lsd1* ko ctrl and *Lsd1* ko ctrl^BMT^ combined, n = 11,024 cells), **B** *Lsd1* ei (n = 2 mice, n = 2580 cells), **C** *Lsd1* ko (n = 2 mice, n = 6352 cells), **D** *Lsd1* ko^BMT^ (n = 2 mice, n = 8913 cells). **E** + **F** and **I** UMAP embeddings of scVI integrations for HSPCs as well as monocytic and granulocytic lineages across all genotypes, colored by: **E** genotype, **F** reference or predicted cell type („-like“ denotates aberrant cells). **G** + **H** Volcano plots of differential gene expression analysis on aggregated pseudo-bulk counts. The dashed red line represents a significance threshold of *p*_adj_ = 0.05. **G** *Lsd1* ko and ko^BMT^ HSPCs vs. control HSPCs, **H** *Lsd1* ei HSPCs vs. control HSPCs. **I** log-normalized *Prtn3* expression levels. **J** **+** **K** Heatmaps depicting the top 20 differentially expressed genes between **J** promonocytes or **K** monocytes and their aberrant counterparts, determined by Wilcoxon rank-sum test, after filtering for genes with at least 25% expression. Colors represent z-scaled log-transformed counts, and color scales have been capped at q5 (lower bound) and q95 (upper bound) for each heatmap to accommodate outliers.

To investigate the molecular mechanisms by which LSD1 depletion but not its enzymatic inactivation causes myeloid skewing and maturation arrest, we performed single-cell RNA-sequencing (scRNA-seq) of *Lsd1* ko, *Lsd1* ko ctrl, *Lsd1* ei, *Lsd1* ei ctrl, *Lsd1* ko^BMT^, and *Lsd1* ko ctrl^BMT^ total BM (Fig. 3). We did not include *Lsd1* ei^BMT^, because these mice only displayed a mild hematopoietic phenotype (Fig. 2). In a first step, we harmonized the gene expression data from *n* = 7 control mice (*Lsd1* ei ctrl, *Lsd1* ko ctrl, and *Lsd1* ko^BMT^ ctrl) and assigned reference cell labels to the generated clusters (Fig. 3A and Supplementary Fig. 5 and 6 as well as Supplemental Table 1). De novo clustering of *Lsd1* ei, *Lsd1* ko, and *Lsd1* ko^BMT^ mice revealed a striking difference in cell type diversity between *Lsd1* ei mice on the one hand and *Lsd1* ko and *Lsd1* ko^BMT^ mice on the other (Fig. 3B–D). Corroborating our data shown above that loss of LSD1 enzymatic activity does not substantially affect hematopoiesis, *Lsd1* ei BM includes all lineages as well as all stages of myeloid maturation, ranging from HSPCs through committed progenitors (GMP) to terminally differentiated neutrophils (Fig. 3B). In contrast, *Lsd1* ko and *Lsd1* ko^BMT^ BM is monomorphic, containing mainly myeloid forms (Fig. 3C, D).

Integration of cells from all genotypes revealed that the *Lsd1* ko and *Lsd1*^koBMT^ cells form common, distinct clusters (Fig. 3E), determined by cell type prediction to display features similar to GMP, promonocytes, monocytes, and macrophages (here termed “GMP-like”, “promonocyte-like”, “monocyte-like”, and “macrophage-like”, Fig. 3F). Surprisingly, the HSPCs of all genotypes nonetheless form a shared cluster. Because we hypothesized that the striking myeloid differentiation bias observed in *Lsd1* ko and *Lsd1* ko^BMT^ cells originates in altered HSPC gene expression, we performed differential gene expression analysis between *Lsd1* depleted and control stem and progenitor cells (Fig. 3G). When compared to control HSPCs, both primary and transplanted *Lsd1 ko* cells overexpress myeloid precursor-specific genes (including *Prtn3, Elane*, and *Gfi1*, Fig. 3G). Strikingly, this is not the case in *Lsd1* ei HSPCs (Fig. 3H). The expression of *Prtn3*, already significantly increased in *Lsd1* ko and ko^BMT^ HSPCs, is likewise overexpressed in the more mature *Lsd1* depleted myeloid progeny (Fig. 3I–K). These data show that all *Lsd1* deleted BM cells, both those in primary *Lsd1* ko BM and those in BM generated following transplantation (*Lsd1* ko^BMT^ BM), display elevated *Prtn3* expression and show a myeloid signature. By single-cell gene expression, these data thus corroborate the overwhelmingly myeloid phenotype witnessed by morphology and immunophenotype (Fig. 1A–F; Supplementary Fig. 3E–J; Fig. 2D–I, and Supplementary Fig. 4B–G). Our data show that absence of the LSD1 protein, but not mere absence of its enzymatic inactivity, leads to elevated expression of myeloid-specific genes and an almost exclusive commitment to the myeloid lineage with a concomitant decrease in lymphoid output (Supplementary Fig. 7A–D). As the phenotype is transplantable, it is generated by absence of *Lsd1* in HSPCs, which causes a bias towards myeloid differentiation as well as a maturation arrest at a GMP-like state.

### LSD1 regulates GFI1 binding to the *PRTN3* locus

**Fig. 4:**
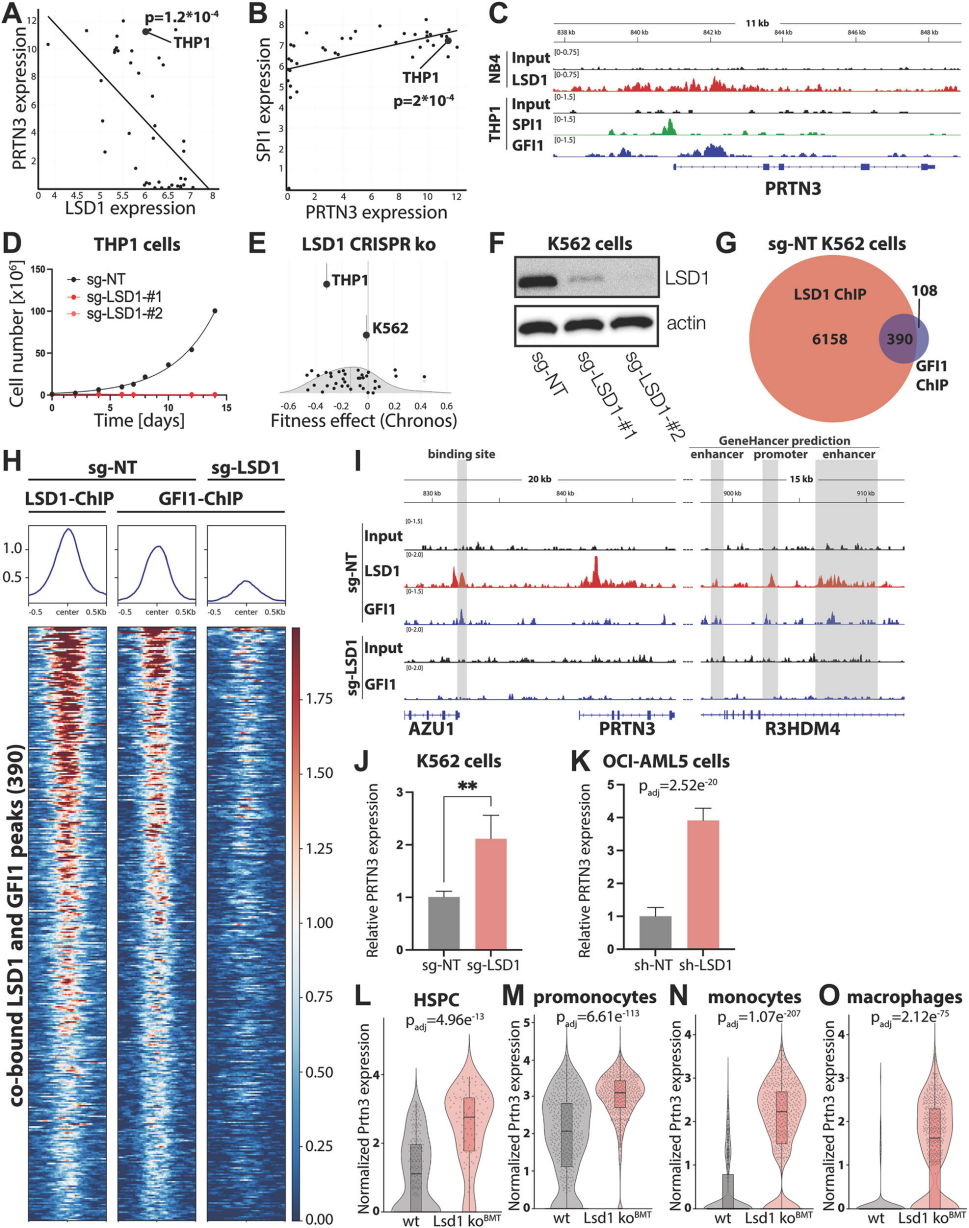
LSD1 regulates GFI1 binding to regulatory elements at the *PRTN3* locus. **A** + **B** Correlation of gene expression in myeloid cell lines by DepMap Public 23Q2 (https://depmap.org/portal/). The monocytic cell line THP1 is highlighted. Expression is shown as log_2_ (TPM + 1). Significance calculated via the DepMap linear regression model using two class comparison **A** correlation between *LSD1* and *PRTN3*. **B** correlation between *SPI1* and *PRTN3*. **C** Integrative Genomics Viewer (IGV) tracks of LSD1 (red), SPI1 (green), and GFI1 (blue) ChIP-seqs. Input controls (black). The *PRTN3* locus is shown. The SPI1 and GFI1 ChIP-seqs were performed in THP1 cells. LSD1 ChIP-seq by Ravasio et al. on NB4 cells [5]. **D** Proliferation of Cas9-expressing THP1 cells following transduction with sgRNAs against a non-targeting region (NT) or against two different sites in *LSD1* (LSD1-#1 and LSD1-#2). **E** Effect of *LSD1* CRISPR ko on the fitness of myeloid cell lines, data from DepMap Public 24Q2 (Chronos) [29]. THP1 and K562 cells are highlighted. **F** Validation of the *LSD1* CRISPR ko in K562 cells by western blot. **G** Venn diagram showing the number of LSD1 and GFI1 binding sites and their overlap, as determined by ChIP-seq in sg-NT K562 cells. **H** Heat map of regions bound by both LSD1 and GFI1 in non-targeted K562 cells (sg-NT, left). These same regions were interrogated for GFI1 binding in LSD1-depleted K562 cells (sgLSD1, right). **I** IGV tracks of LSD1 and GFI1 ChIP-seqs in non-targeted K562 cells (sg-NT) as well as in LSD1-depleted K562 cells (sgLSD1). *PRTN3* regulatory elements, highlighted in gray, were identified either by LSD1 binding or by prediction using the GeneHancer algorithm (from upstream to downstream: GH19J000898, GH19J000909, and GH19J000906, respectively) [30]. **J** Relative *PRTN3* expression in non-targeted (sg-NT) and LSD1-depleted (sgLSD1) K562 cells quantified by qRT-PCR. n = 4 per condition. Statistical testing was performed using Student’s *t* test. **p < 0.01. **K** Relative *PRTN3* expression in OC1-AML5 cells, transduced with a non-targeting shRNA (sh-NT) or an shRNA targeting *LSD1* (shLSD1), quantified by RNA-seq, data mined from Fiscus et al*.* [31]. *Prtn3* expression determined by scRNA-seq in **L** HSPCs, **M** promonocytes, **N** monocytes, and **O** macrophages. **K** Significance calculated using DESeq2 (Wald test). **L**–**O** Significance calculated using Wilcoxon rank sum test.

We investigated the molecular mechanism causing myeloid lineage restriction in *Lsd1* ko hematopoiesis. In a large set of myeloid cell lines, *PRTN3* and *LSD1* expression are negatively correlated (Fig. 4A), supporting our observation that LSD1 deletion increases *PRTN3* expression. Conversely, expression of *PRTN3* and the transcription factor *SPI1* are positively correlated (Fig. 4B), suggesting that SPI1 may regulate *PRTN3* expression. SPI1 has previously been shown to bind the *PRTN3* promoter in gel shift assays [18]. To assess SPI1 binding, we performed a SPI1 ChIP-seq in THP1 cells, a monocytic cell line expressing very high *PRTN3* levels (Fig. 4A, B). SPI1 bound the *PRTN3* promoter region in THP1 cells (Fig. 4C), supporting the hypothesis that it positively regulates *PRTN3* expression.

LSD1 and the transcriptional repressor GFI1 frequently co-localize at target sites, where GFI1 counteracts transcriptional activation by SPI1 [14]. By ChIP-seq, we were able to show that in THP1 cells GFI1 binds the *PRTN3* promoter near the SPI1 binding site (Fig. 4C). Moreover, using NB4 cells, Ravasio et al. [5] have demonstrated LSD1 binding to the *PRTN3* locus (Fig. 4C).

In silico analyses show that SPI1 and LSD1 also bind the *Prtn3* locus in primary murine cells (Supplementary Fig. 8A). SPI1, GFI1, and LSD1 likewise co-localized at previously described common target genes, *CD11B (ITGAM), GFI1*, and *CD86* (Supplementary Fig. 8B). Motif analyses provide further support of common regulatory activity, as SPI1 and GFI1 motifs are highly enriched among LSD1 ChIP-seq peaks (Supplementary Fig. 8C).

In order to test whether GFI1 DNA binding requires presence of the LSD1 protein, we deleted *LSD1* in THP1 cells using CRISPR-Cas9 genome editing. Two different *LSD1*-targeted sgRNA sequences were used. While a non-targeting sgRNA had no measurable effect on THP1 viability, both *LSD1*-targeting sgRNAs severely impaired THP1 cell proliferation and viability so that the cultures could not be maintained (Fig. 4D). Mining the DepMap Public 24Q2 database using the CHRONOS algorithm [29], which infers gene knock out fitness effects based on an explicit model of proliferation dynamics, confirmed that THP1 cells are highly sensitive to *LSD1* depletion (Fig. 4E). The same algorithm predicted that K562 viability is not affected by a loss of LSD1 (Fig. 4E), and we therefore chose this line as an appropriate model to assess the effect of *LSD1* depletion. Using the same *LSD1* sgRNAs, we obtained two independent CRISPR-edited, LSD1-depleted K562 lines (K562 sg-LSD1-#1 and sg-LSD1-#2). LSD1 protein expression was decreased by over 90% in both lines (Fig. 4F). As sg-LSD1-#2, was slightly more efficient, we conducted the subsequent experiments using this *LSD1*-depleted K562 cell line, as well as the non-targeting control (K562 sg-NT).

ChIP-seq analysis of LSD1 and GFI1 in control K562 cells, transduced with a non-targeting sgRNA, (sg-NT), revealed that the majority of GFI1-bound sites are also occupied by LSD1 (Fig. 4G and Supplementary Fig. 8D). A total of 390 GFI1-LSD1 co-occupied sites were identified (Fig. 4G). The low number of GFI1 bound sites is due to technical limitations of the available GFI1 antibodies, which are very challenging in ChIP-seq assays (T. Möröy, personal communication). Nonetheless, we observe that a large majority of the GFI1-bound sites identified were shared with LSD1. Assessing the 390 co-occupied sites in LSD1-depleted cells showed a drastic reduction of GFI1 binding in absence of the LSD1 protein (Fig. 4H). Absence of GFI1 binding following LSD1 depletion was also evident when all LSD1 occupied sites were interrogated for GFI1 co-binding (Supplementary Fig. 8E).

GFI1 binding at the *PRTN3* locus was likewise affected by *LSD1* depletion (Fig. 4I). In control non-targeted K562 cells, four sites in the vicinity of the *PRTN3* gene were co-occupied by GFI1 and LSD1 (Fig. 4I, sg-NT, top tracks, co-bound sites shaded in gray). Three of these four sites are predicted to be enhancer or promoter sequences by the GeneHancer algorithm [30]. One site, within intron 1 of the *PRTN3* gene, which was co-occupied by LSD1 and GFI1 in THP1 cells (Fig. 4C) is occupied by LSD1 only in K562 cells. Upon *LSD1* depletion, GFI1 binding at all four previously co-occupied sites in the *PRTN3* locus was lost (sg-LSD1, Fig. 4I, lower tracks). These data strongly suggest that presence of the LSD1 protein is required for binding of GFI1 to its cognate sites.

Concomitant with a loss of GFI1 binding, *LSD1* depletion increased *PRTN3* expression in K562 cells (Fig. 4J). Mining data obtained by Fiskus et al. showed that *LSD1* depletion in OCI-AML5 cells likewise increased *PRTN3* expression (Fig. 4K) [31]. As shown above in both *Lsd1* ko genotypes analyzed concurrently (Fig. 3G), *Prtn3* expression is also significantly increased when pure *Lsd1* ko^BMT^ HSPCs are compared to their wt counterparts (Fig. 4L). Moreover, *Prtn3* expression is significantly increased in *Lsd1* ko^BMT^ promonocytes, monocytes, and macrophages (Fig. 4M–O). Taken together, these data, obtained in three different hematopoietic contexts, strongly suggest that LSD1 inhibits *Prtn3* expression at least in part by enabling binding of the GFI1 repressor to the locus.

### *Prtn3* depletion in *Lsd1* ko mice restores impaired myeloid differentiation and reverses expansion of both the myeloid and the stem cell compartments

**Fig. 5:**
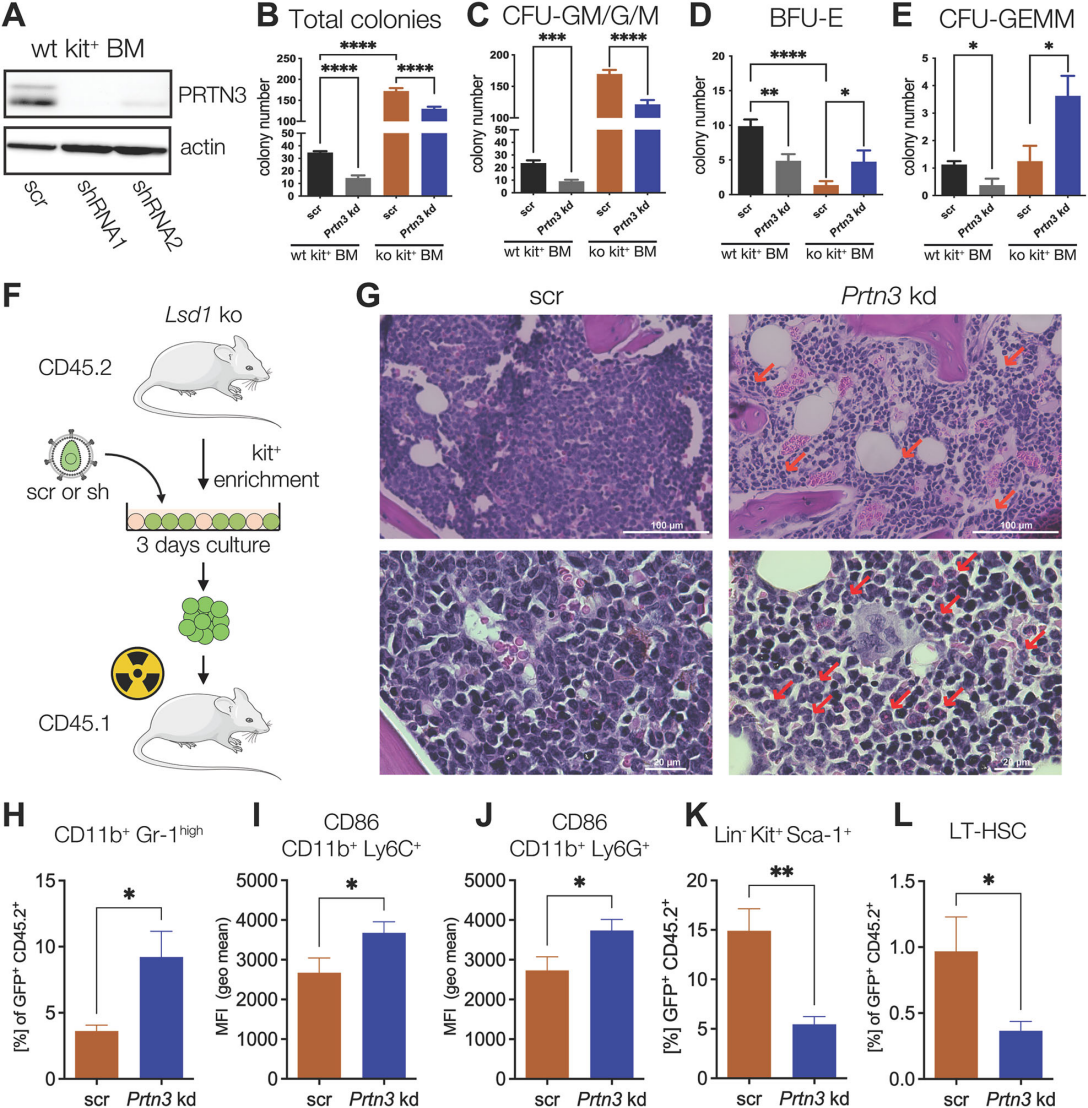
*Prtn3* knockdown decreases myeloid colony formation, induces myeloid cell differentiation, and reduces stem and progenitor cells in the *Lsd1* ko context. **A** Knockdown validation (shRNA) of *Prtn3* in kit^+^ bone marrow (BM) cells 48 h after transduction by western blot. **B**–**E** Colony counts of wild-type (wt) and *Lsd1* ko kit^+^ BM transduced with scrambled-shRNA control virus (scr) or shRNA1 against *Prtn3*: *n* = 4 per group and genotype. Statistical testing was performed using Student’s *t* tests. *p < 0.05, **p < 0.01, ***p < 0.001, ****p < 0.0001. **B** total number of colonies **C** CFU-GM, CFU-G and CFU-M, **D** BFU-E, **E** CFU-GEMM. **F**–**L** *Prtn3* in vivo knockdown in the *Lsd1* ko context. **F** Experimental setup: kit enriched CD45.2 ko BM was transduced with either scr or shRNA1. Three days later, GFP-positive cells were transplanted into lethally irradiated CD45.1 recipient animals. The panel was designed with Smart Servier. **G**–**L** Mice were analyzed three weeks after transplantation. **G** Histopathological BM slides of mice transplanted with *Lsd1* ko kit^+^ BM transduced either with a scr control (left) or a knockdown for *Prtn3* (right). 200x magnification (top), 1000x magnification (bottom). Red arrows: metamyelocytes. **H**–**L** Flow cytometry analyses of the BM. Statistical analyses were conducted using Student’s *t* tests. *p < 0.05, **p < 0.01. n = 4–5 per condition. **H** CD11b^+^ Gr-1^high^ myeloid cell frequency in GFP^+^ CD45.2^+^ BM (**I** + **J**) Mean fluorescence intensity of **I** CD86 of CD11b^+^ Ly6C^+^ and **J** CD11b^+^ Ly6G^+^ myeloid cells. **K** Frequency of lineage negative kit^+^ sca-1^+^ (LSK) GFP^+^ CD45.2^+^ single cells. **L** Frequency of long-term HSCs (LT-HSCs) GFP^+^ CD45^.^2^+^ single cells.

To assess whether normalizing *Prtn3* expression can reduce the myeloid expansion observed following *Lsd1* deletion, we depleted *Prtn3* expression using RNAi. We validated the efficacy of two different *Prtn3*-targeted shRNA sequences by transduction of wt murine kit^+^ BM cells, followed by western blotting and qPCR. Both sequences depleted *Prtn3* expression by over 90% (Fig. 5A and Supplementary Fig. 9A), and we used shRNA1 henceforth. Subsequently, we assayed the effect of *Prtn3* depletion on colony formation by WT and *Lsd1* ko kit^+^ BM cells, transduced with viruses expressing either an shRNA against *Prtn3* or a scrambled (scr) control (Fig. 5B–E). *Lsd1* ko kit^+^ BM cells formed significantly more colonies in vitro than WT control cells (Fig. 5B). The observed increase in colony counts resulted from a stark increase in myeloid colony formation (colony-forming unit (CFU)-GM, CFU-G, and CFU-M) by the *Lsd1* ko kit^+^ BM cells, confirming the extensive myeloid bias (Fig. 5C and Supplementary Fig. 10). In both genotypes, *Prtn3* depletion decreased the total number of colonies formed (Fig. 5B). Moreover, the proportion of myeloid colonies decreased significantly upon *Prtn3* depletion, supporting our hypothesis that overexpression of *Prtn3* following loss of *Lsd1* contributes to myeloid expansion (Fig. 5C). Fittingly, the number of erythroid colonies, (BFU-E, burst-forming-units erythroid) was significantly reduced in *Lsd1* ko BM and this was partially rescued by *Prtn3* knockdown (Fig. 5D), suggesting that elevated *Prtn3* levels promote the lineage bias observed upon loss of *Lsd1*.

**Fig. 6:**
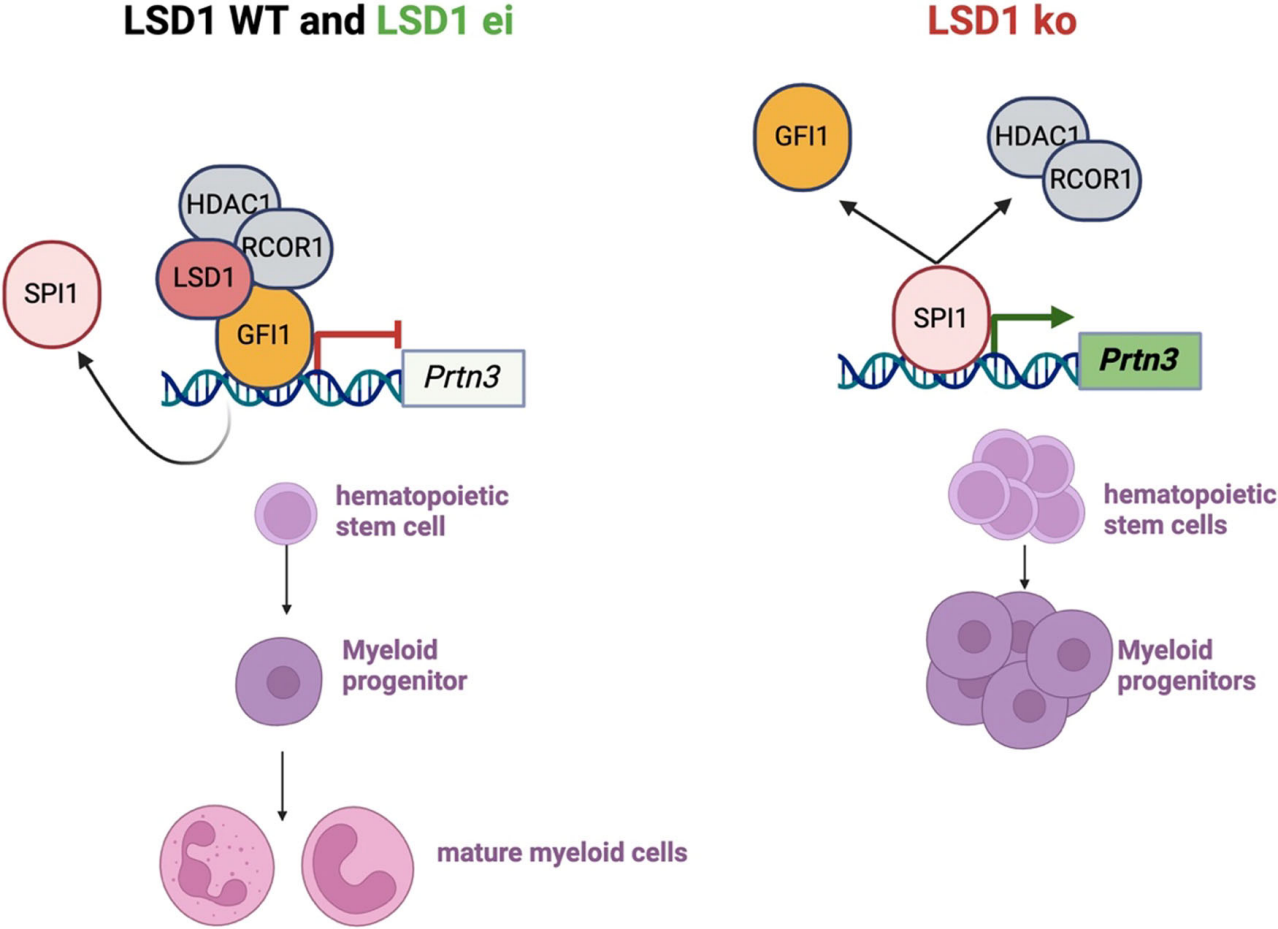
Proposed mechanism: In the presence of LSD1, GFI1 represses the expression of *Prtn3*, thereby regulating stem cell expansion and myeloid cell differentiation. In the absence of LSD1 protein, GFI1 cannot repress *Prtn3* expression, and SPI1 drives sustained *Prtn3* overexpression, leading to the expansion of hematopoietic stem and myeloid progenitor cells and a lack of mature myeloid cells. Figure created with BioRender.com.

We subsequently depleted *Prtn3 expression* in *Lsd1* ko kit^+^ BM and transplanted it into lethally irradiated recipient mice (Fig. 5F). Three weeks after transplantation, *Prtn3* expression in the BM remained suppressed by almost 90% (Supplementary Fig. 9B). At this time, *Lsd1* ko, *Prtn3* knockdown animals displayed significantly lower white blood cell counts and increases in the still very low platelet counts with no changes in the differential blood counts or the hemoglobin levels (Supplementary Fig. 9C–J). Histopathological and cytomorphological analyses showed a release of the myeloid differentiation block by *Prtn3* depletion, with more mature myeloid forms appearing in the BM (metamyelocytes, red arrows, Fig. 5G and Supplementary Fig. 9K). Concomitantly, more mature myeloid cells (CD11b^+^, Gr-1^high^) were detected by flow cytometry analyses (Fig. 5H). This included increased phenotypic maturation into CD86^+^ cells (CD11b^+^ Ly6C^+^ and CD11b^+^ Ly6G^+^, Fig. 5I+J). The increase in stem and progenitor cells observed in the *Lsd1* ko context was likewise reversed by *Prtn3* depletion, witnessed by decreased stem and progenitor cells (LSK) and decreased LT-HSCs (Fig. 5K+L).

In summary, we show that knockdown of the aberrantly increased *Prtn3* expression in the *Lsd1* ko context is sufficient to restore myeloid cell differentiation and to decrease expansion of the stem cell compartment. From our data we propose the following molecular mechanism: in the presence of LSD1, GFI1 binds the *Prtn3* promoter together with LSD1 and represses *Prtn3* expression. In this condition, stem cell and progenitor expansion is restrained, and myeloid maturation remains intact. In absence of the LSD1 protein, GFI1 cannot bind its cognate site and fails to repress *Prtn3*. Consequently, sustained SPI1 transcriptional activity drives *Prtn3* overexpression, leading to the expansion of hematopoietic stem cells and myeloid progenitors, myeloid lineage restriction, as well as to impaired myeloid maturation (Fig. 6).

## Discussion

A quintessential role for LSD1 in hematopoietic stem cell differentiation has previously been described [12, 13]. More recently, it has become clear that LSD1 functions in different ways, both enzymatically as a demethylase and structurally, by serving as a scaffold for protein-protein interactions. Several studies have identified non-enzymatic, pro-leukemogenic roles of LSD1 in the context of AML [1, 5, 32]. However, the role of LSD1 in healthy hematopoiesis and the molecular mechanisms by which LSD1 affects healthy hematopoiesis are not understood in detail. We studied two unique murine models in parallel to address this question, one in which the entire LSD1 protein is deleted and a second which selectively abrogates LSD1 enzymatic activity through point mutations that leave the structural protein intact.

Complete loss of the LSD1 protein in all tissue types led to altered lineage distribution in the BM, resulting in a myeloid predominance accompanied by a maturation block as well as stem cell expansion (Fig. 1). These data are similar to the phenotype observed upon depletion of *Lsd1* by RNAi [13]. All of these changes, the myeloid lineage bias, the myeloid maturation block, and the HSC expansion are cell intrinsic, as they are recapitulated upon transplantation of *Lsd1* deficient HSCs into lethally irradiated recipients (Fig. 2). Importantly, these changes are brought about by loss of the LSD1 protein, not by abrogation of its enzymatic activity, as neither animals in which LSD1 was enzymatically inactive in all cells, nor secondary recipients of LSD1 enzymatically inactive HSCs showed these phenotypes. Hence, LSD1 scaffolding function, not its demethylase activity, is required for HSC homeostasis, for balanced HSC lineage commitment as well as for complete myeloid maturation.

Mechanistically, we showed that the cell-intrinsic myeloid bias as well as the myeloid maturation block result from the de-repression of myeloid regulatory genes, including the protease *Prtn3* (Fig. 3). We provide evidence that *Prtn3* is a direct target of both LSD1 and GFI1 (Fig. 4). These two transcriptional repressors are well-described cooperators and vital for the regulation of gene expression throughout HSC emergence and differentiation [11, 33]. GFI1 acts as a repressor, frequently antagonizing activity of the transcription factor SPI1, as the two proteins often co-regulate myeloid target genes [4].

We propose a model in which, in the absence of LSD1, GFI1 is no longer tethered to these promoters, allowing unhindered binding and activation by SPI1 (Fig. 6). In its support, we provide experimental evidence for the absence of GFI1 chromatin binding in LSD1-depleted cells (Fig. 4H and Supplementary Fig. 8D). Moreover, *Lsd1* depletion but not its enzymatic inactivation strongly upregulated *Prtn3* expression in hematopoietic stem and progenitor cells (Fig. 3G, H). Importantly, RNAi-mediated repression of the elevated *Prtn3* levels in *Lsd1* ko cells reduced aberrant stem cell expansion (Fig. 5K, L) and restored myeloid maturation (Fig. 5H–J). In our model, we therefore propose that aberrant *Prtn3* expression in absence of LSD1 scaffolding function contributes to stem cell expansion, myeloid lineage restriction as well as myeloid maturation arrest.

LSD1 has been reported to be overexpressed in myeloid malignancies and proposed as a therapeutic target [1–8]. Interestingly, we show here that the effect of LSD1 inhibition in healthy hematopoietic cells is different to that in AML cells. Pharmacological LSD1 inhibition in AML cells upregulates CD11b and induces myeloid differentiation [8]. While we also recapitulate CD11b upregulation by LSD1 deletion, we show here that in healthy hematopoiesis loss of LSD1 blocks myeloid differentiation, arresting cells at the CD11b^+^ Gr-1^low^ stage (Fig. 2). Results similar to ours were in part observed using RNAi-mediated *Lsd1* knockdown [13]. However, *Lsd1* depletion by Sprüssel et al. only reached 40–50%, leaving the possibility that residual LSD1 protein function affected the phenotype. Similarly, Kerenyi and colleagues did not address myeloid maturation in detail [12]. Our data therefore consolidate the role of LSD1 protein scaffolding function in HSC homeostasis, lineage commitment, and myeloid maturation, and provide definite evidence for the difference in LSD1 function between malignant AML cells and healthy hematopoiesis.

In addition to HSC homeostasis and myelopoiesis, it has been shown that LSD1 recruitment is required for normal megakaryo- and thrombopoiesis [11]. However, it is not understood whether both the scaffolding function and the enzymatic activity of LSD1 are required for platelet formation. This is in part due to the fact that most research perturbing LSD1 has been performed in the context of AML, with a focus on the disruption of LSD1 protein complexes [11, 34]. It has been shown that GFI1B, which, in contrast to the myeloid progenitor-specific GFI1, is expressed in HSCs and megakaryocyte progenitors, is required for the development of megakaryocytes [34]. GFI1B recruits the CoREST complex, including LSD1, RCOR1, and HDAC1/2, to its target genes [11]. Epigenetic regulation of healthy megakaryopoiesis by GFI1B is therefore mediated in part by LSD1 serving as a scaffold for the assembly of protein complexes.

While the structural role of the LSD1/GFI1B-complex in mediating gene regulation during megakaryopoiesis has thus been established, the contribution of LSD1 enzymatic activity has not been investigated. Here, we show that *Lsd1* ei^BMT^ mice, in which LSD1 enzymatic activity is deleted solely in the bone marrow, contain reduced platelet numbers (Fig. 2) and fewer megakaryocytes in the BM (data not shown). This suggests that LSD1 enzymatic function is required for megakaryo- and thrombopoiesis. Further studies will be required to unravel the mechanisms behind the observed phenotype and assess the extent to which the LSD1 demethylase activity contributes to normal megakaryo- and thrombopoiesis.

## Materials and methods

### Ethics approval

All methods were performed in accordance with the relevant guidelines and regulations. Experiments were performed in accordance with committee-approved animal protocols (Environment and Consumer Protection of the state Baden-Württemberg, Germany, G-14/47 and G-20/102). All animals were kept under specific pathogen-free conditions at the research mouse facility of the University Medical Center Freiburg. A power analysis was conducted using G*Power (version 3.1) to approximate the sample size. Based on an expected effect size of d = 0.95, α = 0.05, and power = 0.8–0.95, 8–12 animals per group were required. Randomization and blinding were not applicable.

### *Lsd1* mouse models

Homozygous conditional *Lsd1* knockout mice (CreER^T2^, *Lsd1*^*tm1Schüle*^) [24] and enzymatic inactive mice (CreER^T2^, *Lsd1*^*K661A, W752A, Y762S*^) [25] were used to investigate the *Lsd1*-dependent phenotype in vivo. Cre expression was induced by continuous *i.p*. tamoxifen injections (5x/week, 1 mg/injection in 100 μl corn oil with 10% ethanol), starting between 8 and 18 weeks of age. Female mice were used exclusively for analysis of the total body ko and ei (Fig. 1). *Lsd1* ko and *Lsd1* ko ctrl mice were sacrificed for final analysis when the *Lsd1* ko mice became moribund, on average two weeks after the initiation of tamoxifen treatment. *Lsd1* ei and *Lsd1* ei ctrl mice were observed for eight weeks before sacrifice. To analyze the isolated hematological phenotype, bone marrow cells from induced *Lsd1* ko and ei mice (CD45.2) were transplanted into lethally irradiated (2 × 5.5 Gy) CD45.1 female recipient mice, via the retrobulbar technique. These mice were termed *Lsd1* ko^BMT^ and *Lsd1* ei^BMT^.

### Complete blood cell counts (CBC)

Peripheral blood samples from mice were taken via puncture of retrobulbar veins with a heparin-coated 10 μl capillary and collected in heparin-coated 300 μl microvette tubes. Subsequent complete blood cell count analyses were performed on an Animal Blood Counter Plus (Scil Vet).

### Flow cytometry

Flow cytometry experiments were performed on a BD FACS Fortessa. Lymphoid, erythroid, and myeloid cells were detected by staining peripheral blood and bone marrow for B220 (BioLegend, clone RA3-6B2), CD3 (Thermo Scientific, clone 145-2C11), Gr*-*1 (BioLegend, clone RB6-8C5), CD11b (BioLegend, clone M1/70), Ter-119 (BioLegend, clone TER-119) and CD71 (BioLegend, clone R17217).

Stem and progenitor cells were detected by staining with a cocktail against lineage markers (BioLegend, B220, CD3, Gr1, Mac1, and Ter119) and staining for c-Kit (eBioscience, clone 2B8), Sca1 (BioLegend, clone D7), CD34 (BioLegend, clone MEC14.7), Fc-γ-II/III-R (eBioscience, clone 93), Thy1.1 (BioLegend, clone OX7), and Flt3 (eBioscience, clone A2F10).

Antibodies against CD45.1 (BioLegend, clone A20) and CD45.2 (BioLegend, clone 104) were used to distinguish between donor and recipient-derived cells. Gating strategies were determined by fluorescence minus one staining as previously described [35]. Antibodies against Ly6G (BioLegend, clone 1A8), Ly6C (BioLegend, clone HK1.4), and CD86 (BioLegend, clone GL-1) were used to detect myeloid subpopulations.

### Isolation of kit^+^ BM cells

Whole bone marrow cells were obtained in PBS containing 3% FCS and 1% Penicillin/Streptomycin. Erythrolysis was performed using the BD lysis buffer (BD, Biosciences, 555899). Kit^+^ bone marrow cells were isolated using CD117 MicroBeads (Miltenyi Biotec, 130097146) and LS columns (Miltenyi Biotec, 130042401) according to the manufacturer’s instructions. Kit^+^ cells were cultivated in SFEM medium (Stemcell Technologies, 09650) supplemented with 10 ng/ml mIL3 (PeproTech, 213-13), 10 ng/ml mIL6 (PeproTech, 216-16), and 50 ng/ml mSCF (PeproTech, 250-03).

### Lentiviral *Prtn3* knockdown

A modified pLeGO-iG-U6 vector (termed iG-hU6) was used for lentiviral transduction as previously described [36]. To study *Prtn3* depletion, *Prtn3* shRNAs and a scrambled control shRNA were introduced into WT kit^+^ and ko kit^+^ BM cells using the iG-hU6-sh-Prtn3-#1 (GTCAGGTCTTCCAGAACAATT), the iG-hU6-sh-Prtn3-#2 (CCCTTGATCTGCAATGGCATT), and the iG-hU6-scr (ATGTTCTACGCTCAATGCGG) constructs.

### Lentiviral *LSD1* CRISPR-Cas9 gene editing

THP1 and K562 cells were obtained and cultured following the recommendations from DMSZ (German Collection of Microorganisms and Cell Cultures). For CRISPR-Cas9 gene editing, THP1 and K562 cells were initially transduced with pLenti-Cas9-P2A-Puro (Addgene #110837), and cells gaining stable Cas9 expression were selected by culture in 2 μg/ml Puromycine. In a second step, *LSD1* sgRNAs or a non-targeting sgRNA, expressed by the pLKO5.sgRNA.EFS.GFP vector (Addgene #57822) was introduced into Cas9-expressing cells. The following sequences were chosen: sg-LSD1-#1 (TCATCCGGTCATGAGGAAGT); sg-LSD1-#2 (AGCTGATCTTGGAGCCATGG); sg-NT(ACGGAGGCTAAGCGTCGCAA). *LSD1* ko efficiency was determined by Western Blot.

### Quantitative real-time PCR (qRT-PCR)

RNA isolation and reverse transcription were performed using RNAeasy (QIAgen, 74104) and SuperScript™ II Reverse Transcriptase (Invitrogen, 18064022). Expression of human *PRTN3* (Thermo Fisher Scientific, Waltham, Massachusetts, USA, Hs01553330_m1) or murine *Prtn3* (Mm00478323_m1) was determined in cDNA derived from K562 or murine BM cells, respectively. β-2-microglobulin (*B2m*) was used as housekeeping gene (Thermo Fisher Scientific, Assay on Demand). Data were analyzed using the ΔΔCT method.

### Sanger sequencing

Sanger sequencing was performed to interrogate the efficiency and permanence of Tamoxifen-induced CRE-mediated recombination in *Lsd1* ei and *Lsd1* ei ctrl mice. RNA isolation and reverse transcription were performed as described above. A fragment of the *Lsd1* cDNA was amplified using the *Lsd1*-cDNA-Seq-FP forward primer (5′-TTGCTGTGAACACACGTTCC-3′) and *Lsd1*-cDNA-Seq-RP reverse primer (5′-AGAGTCTTGGGATTGGCTGTG-3′) primers and the KAPA HiFi PCR Kit (Roche, KK2102). Following purification with the DNA Clean & Concentrator-5 Kit (Zymo, D4014), the resulting fragment was subjected to sequencing.

### Western blotting

Murine BM cells or K562 cells were lysed in RIPA buffer (150 mM NaCl, 1% Triton X-100, 0.5% NaDoc, 0.1% SDS, and 50 mM Tris, pH 8.0) for 30 min on ice. Cell debris was removed by centrifugation. Protein concentrations were estimated by Lowry assay (Bio-Rad, 5000112). Immunoblotting was performed as previously described [37]. Primary antibodies: anti*-*PRTN3 (LSBio, C692449), anti-LSD1 (produced in the Schüle Laboratory) [38], and anti*-*beta*-*actin (Sigma-Aldrich, A5441). Secondary antibodies: anti-rabbit (GE Healthcare, NA934V) and anti-mouse (GE Healthcare, NA931V) IgG HRP.

### Colony assays

Colony assays were performed as previously described [39]. Bone marrow cells were seeded in methylcellulose media supplemented with SCF, IL3, IL6, and EPO (STEMCELL Technologies, 09650). On days 10-14, CFU-GEMM, BFU-E, CFU-GM, CFU-G, and CFU-M colonies were scored after staining with benzidine.

### Histopathological analysis

Femur and spleen samples were fixed in 4% formalin overnight. Femora were consecutively decalcified in 10% buffered ethylene-diamine tetra-acetic acid (EDTA, pH 7.2). Organs were paraffin-embedded as previously described [35], and sections were stained with hematoxylin and eosin.

### Cytospins

For cytospins, 2000–40,000 BM cells were centrifuged in a Cytospin 3 centrifuge (Shandon, Runcorn, GB) for three minutes at 800 rpm at room temperature.

### May-Grünwald-Giemsa (MGG) staining

Peripheral blood smears as well as PB and BM cytospins were stained for 5 min with May-Grünwald solution (Merck, Rahway, New Jersey, Cat. No. 1014240500). After washing with buffered water (pH 6.8), Giemsa counter-staining was performed for 15 min (Giemsa diluted 1:15 in buffered water pH 6.8), (Merck, Cat. No. 1092040500). Following a final wash, slides were air-dried.

### Chromatin immunoprecipitation sequencing (ChIP-seq)

ChIP-seq was used to interrogate DNA binding of GFI1, SPI1, and LSD1 in THP1 and K562 cells. GFI1, SPI1, and LSD1 ChIPs were performed as described in Helness et al. [40]. Per ChIP, 10 million cells were fixed with 1.5 mM DSG and 1% formaldehyde before quenching with 125 mM glycine. After cell lysis and nuclei extraction, chromatin was sheared using a Covaris S220 to generate 100–500 bp fragments. Samples were immunoprecipitated with 5 μg antibody either against GFI1 (R&D Systems, AF3540), SPI1 (Invitrogen, PA5-17505), or LSD1 (produced in the Schüle Laboratory) [38]. Libraries were generated using an NEBNext Ultra II DNA Library Prep Kit (New England Biolabs, E7645).

RELACS (restriction enzyme-based labeling of chromatin in situ) ChIP-seq [41] was used to interrogate the H3K4me1 histone mark. One to three million kit^+^ BM cells from *Lsd1* ei and *Lsd1* ei ctrl mice were fixed with 1% formaldehyde before quenching with 125 mM glycine and snap freezing in liquid nitrogen. Nuclei extraction, chromatin preparation, immunoprecipitation, and library preparation were performed as described in Arrigoni et al. [41]. For immunoprecipitation, an antibody against H3K4me1 (Diagenode, C15410194) was used. ChIP-seq libraries were sequenced on a NovaSeq 6000 to obtain 100 bp paired-end reads.

### Publicly available data used in this work

Publicly available ChIP-seq data [GSE128528, GSE27841, GSE22557, and GSE237192] and RNA-seq data [GSE160303] were obtained from the GEO database and processed as detailed below.

### ChIP-seq data analysis

Analysis of human ChIP-seq data was performed using the following tools and versions: trim-galore v0.6.1, bowtie2 v2.5.4, Picard v3.1.0, macs2 v2.2.9.1, Homer v5.1, deeptools v3.5.5. After removal of low-quality reads and adapter sequences using Trim Galore!, reads were mapped against GRCh38 using bowtie2 with the parameters “-X 1000 --very-sensitive” [42]. Duplicate reads were removed using Picard. High-confidence peaks (p < 10–5) were called with the MACS2 callpeak algorithm [43] using “-f BAMPE --keep-dup all” and blacklisted for low-mappability regions using the GRCh38 ENCODE blacklists [44]. Motif analysis of a 50 bp region surrounding peak summits was performed with the HOMER findMotifsGenome.pl tool [45].

Coverage scores were calculated with deeptools [46] bamCoverage using a bin size of 1 and were normalized to CPM (counts per million) and blacklisted as well. Deeptools computeMatrix and plotHeatmap were used to visualize coverage scores across multiple genomic regions.

Murine LSD1, SPI1, and H3K4me1 ChIP-Seq data were processed on the European usegalaxy.eu [47] servers and mapped to the mm10 genome. The same tools and parameters as for human ChIP-seq analysis were used, except for bowtie2, which was run in “--very sensitive-local” (LSD1, SPI1) or “--local” (H3K4me1) mode and duplicate read removal, which was performed using deeptools alignmentSieve [46]. Coverage scores were computed for 25 bp bins, normalized to RPKM (reads per million mapped reads), and blacklisted for low-mappability regions using the mm10 ENCODE blacklist [44].

### RNA-seq data analysis

RNA-seq data analysis was performed using the European usegalaxy.eu server [47] and Rstudio (R version 4.3.1). Transcript sequence files and gene annotation files were obtained from the GENCODE project (Human release 47) [48]. Reads were trimmed using Trim Galore! and transcripts were quantified using Salmon [49]. Tximport [50] and DESeq2 [51] were used for importing raw counts and differential expression analysis.

### Single-cell RNA sequencing

Viably frozen cells were thawed at 37 °C, resuspended in ice-cold PBS, washed twice, and counted with a LUNA automated cell counter (Logos Biosystems). Single cell capture, reverse transcription, and library preparation were carried out on the Chromium platform (10x Genomics) with the single cell 3ʹ reagent v2 protocol according to the manufacturer’s recommendations using 1000 cells as input per reaction channel. The cDNA libraries were paired-end sequenced (26 bp and 74 bp) on the Illumina HiSeq 4000 system. Raw sequencing data were processed and aligned to the murine genome GRCm39 using the CellRanger pipeline (10x Genomics, version v9.0.0).

### Single-cell RNA sequencing data pre-processing

We applied sample-specific quality control and excluded low-quality cells with mitochondrial read fractions exceeding 5 median absolute deviations (MAD) above the median. This approach preserved cell type composition across samples, including those where cells with low transcriptional complexity (erythrocytes and neutrophils) were abundant. We detected residual ambient RNA from red blood cell lysis and neutrophil degranulation. We addressed this issue by applying SoupX to both the raw and the filtered feature-barcode matrices [52]. Using the autoEstCont() function, we estimated background reads from both empty droplets and non-expressing cell clusters, effectively removing ambient RNA contamination. We identified and flagged doublets using the R/Bioconductor package scDblFinder, ensuring that no cluster was artificially enriched for them [53].

### Single-cell RNA sequencing data analysis

Subsequent data analysis was performed using Scanpy [54] and Seurat V5 [55]. In brief, Scanpy was used to generate log-normalized counts and select n = 3000 highly variable genes (HVGs), excluding cell cycle genes [56]. These HVGs were used for principal component analysis (PCA) with 25–30 components. Euclidean distances in the PC-reduced space were then used to construct a k-nearest neighbor (KNN) graph, and clusters were identified with the Leiden algorithm. Finally, UMAP was applied for further dimensionality reduction and visualization. Seurat was used for visualization in conjunction with the ScCustomize package (10.5281/zenodo.5706430), for marker gene identification via the FindMarkers() function, and for cluster validation at different resolutions using the Louvain algorithm.

For simple integration tasks within the same experimental condition, we used the Python implementation of harmony (https://zenodo.org/badge/latestdoi/229105533) [57]. HVGs were selected from log-normalized counts while specifying mouse donor as the batch key. PCA was then performed, and the resulting principal components were harmonized using a theta value of 1.0. For complex integration tasks across experimental conditions, where the samples had different cell type compositions, we chose deep neural network-based models from scvi-tools [58]. ScVI [59] was used to train models on the raw counts of HVGs, specifying the appropriate batch key and using the following parameters: n_layers = 2, n_latent = 25, gene_likelihood = “zinb”. The resulting integrated latent space then served as the basis for downstream analyses, including UMAP visualizations. To perform label transfers from control (ctrl) to experimental genotypes (ko, ko^BMT^, and ei), we used the same scVI models to train an scANVI model [60], which predicted the closest reference cell type for each cell in the ko, ko^BMT^, and ei conditions.

To identify differentially expressed genes between cell populations we used two approaches. Overall exploration of markers was done using the Seurat FindMarkers() function, and significance was tested using Wilcoxon rank-sum tests. Specifically, for heatmaps displaying top marker genes, we usually filtered for genes expressed in at least 25% of cells. For a more robust identification of DEGs, we performed pseudo-bulk analyses. Briefly, pseudo-samples were created by aggregating counts in the cell population of interest for each mouse donor and were filtered for at least 20 cells per pseudo-sample. Differential gene expression was then tested using the likelihood ratio test implementation of the R/Bioconductor package edgeR (edgeR-LRT) (10.18129/B9.bioc.edgeR) [61].

Differentially expressed genes were used to define cell clusters (Supplementary Figs. 5 and 6).

### Statistical analysis

Unpaired Student *t*-tests were used to determine whether a significant (p < 0.05) difference existed between two groups, unless otherwise stated in the figure legends. Data are presented as mean ± SEM. Values that are two SD from the mean of the group were defined as outliers. Survival analyses were conducted using Log-Rank (Mantel-Cox) test. Analyses were performed using the GraphPad Prism 10 software.

### Material requests

Correspondence regarding material requests other than mouse lines should be addressed to H.F. Staehle. Mouse line requests should be addressed to R. Schüle, the owner of the mouse lines.

## Supplementary information

Supplemental Figures

Supplemental Table 1

Uncropped Western Blots

Publication License BioRender

## Data Availability

All data generated or analyzed during this study are either included in this published article and its supplementary information files or will be made available upon request. The single cell RNA-sequencing and the ChIP-seq data sets generated and analyzed during the current study have been submitted to the GEO database under the accession numbers GSE286396 and GSE286398.

## Code availability

The analysis code, including Jupyter notebooks and R Markdown files necessary to reproduce our analyses, has been hosted in a project-specific GitHub repository: https://github.com/chriskoellerer/Lsd1_ko_scRNAseq.

## References

[1] Cusan M, Cai SF, Mohammad HP, Krivtsov A, Chramiec A, Loizou E, et al. LSD1 inhibition exerts its antileukemic effect by recommissioning PU.1- and C/EBPα-dependent enhancers in AML. Blood. 2018;131:1730–42. 29453291 10.1182/blood-2017-09-807024 PMC5897868

[2] Maes T, Mascaró C, Tirapu I, Estiarte A, Ciceri F, Lunardi S, et al. ORY-1001, a potent and selective covalent KDM1A inhibitor, for the treatment of acute leukemia. Cancer Cell. 2018;33:495–511.e12. 29502954 10.1016/j.ccell.2018.02.002

[3] Mould DP, McGonagle AE, Wiseman DH, Williams EL, Jordan AM. Reversible inhibitors of LSD1 as therapeutic agents in acute myeloid leukemia: clinical significance and progress to date. Med Res Rev. 2015;35:586–618. 25418875 10.1002/med.21334

[4] Barth J, Abou-El-Ardat K, Dalic D, Kurrle N, Maier A-M, Mohr S, et al. LSD1 inhibition by tranylcypromine derivatives interferes with GFI1-mediated repression of PU.1 target genes and induces differentiation in AML. Leukemia. 2019;33:1411–26. 30679800 10.1038/s41375-018-0375-7

[5] Ravasio R, Ceccacci E, Nicosia L, Hosseini A, Rossi PL, Barozzi I, et al. Targeting the scaffolding role of LSD1 (KDM1A) poises acute myeloid leukemia cells for retinoic acid–induced differentiation. Sci Adv. 2020;6:eaax2746. 32284990 10.1126/sciadv.aax2746 PMC7141832

[6] Magliulo D, Bernardi R, Messina S. Lysine-specific demethylase 1A as a promising target in acute myeloid leukemia. Front Oncol. 2018;8:255. 30073149 10.3389/fonc.2018.00255 PMC6060236

[7] Niebel D, Kirfel J, Janzen V, Höller T, Majores M, Gütgemann I. Lysine-specific demethylase 1 (LSD1) in hematopoietic and lymphoid neoplasms. Blood. 2014;124:151–2. 24993879 10.1182/blood-2014-04-569525

[8] Fiskus W, Sharma S, Shah B, Portier BP, Devaraj SGT, Liu K, et al. Highly effective combination of LSD1 (KDM1A) antagonist and pan-histone deacetylase inhibitor against human AML cells. Leukemia. 2014;28:2155–64. 24699304 10.1038/leu.2014.119 PMC4739780

[9] Metzger E, Wissmann M, Yin N, Müller JM, Schneider R, Peters AHFM, et al. LSD1 demethylates repressive histone marks to promote androgen-receptor-dependent transcription. Nature. 2005;437:436–9. 16079795 10.1038/nature04020

[10] Lee MG, Wynder C, Cooch N, Shiekhattar R. An essential role for CoREST in nucleosomal histone 3 lysine 4 demethylation. Nature. 2005;437:432–5. 16079794 10.1038/nature04021

[11] Saleque S, Kim J, Rooke HM, Orkin SH. Epigenetic regulation of hematopoietic differentiation by Gfi-1 and Gfi-1b is mediated by the cofactors CoREST and LSD1. Mol Cell. 2007;27:562–72. 17707228 10.1016/j.molcel.2007.06.039

[12] Kerenyi MA, Shao Z, Hsu Y-J, Guo G, Luc S, O’Brien K, et al. Histone demethylase Lsd1 represses hematopoietic stem and progenitor cell signatures during blood cell maturation. Elife. 2013;2:e00633. 23795291 10.7554/eLife.00633 PMC3687337

[13] Sprüssel A, Schulte JH, Weber S, Necke M, Händschke K, Thor T, et al. Lysine-specific demethylase 1 restricts hematopoietic progenitor proliferation and is essential for terminal differentiation. Leukemia. 2012;26:2039–51. 22699452 10.1038/leu.2012.157

[14] Dahl R, Iyer SR, Owens KS, Cuylear DD, Simon MC. The transcriptional repressor GFI-1 antagonizes PU.1 activity through protein-protein interaction*. J Biol Chem. 2007;282:6473–83. 17197705 10.1074/jbc.M607613200 PMC3218793

[15] Hock H, Hamblen MJ, Rooke HM, Traver D, Bronson RT, Cameron S, et al. Intrinsic requirement for zinc finger transcription factor Gfi-1 in neutrophil differentiation. Immunity. 2003;18:109–20. 12530980 10.1016/s1074-7613(02)00501-0

[16] McKercher SR, Torbett BE, Anderson KL, Henkel GW, Vestal DJ, Baribault H, et al. Targeted disruption of the PU.1 gene results in multiple hematopoietic abnormalities. EMBO J. 1996;15:5647–58. 8896458 PMC452309

[17] Scott EW, Simon MC, Anastasi J, Singh H. Requirement of transcription factor PU.1 in the development of multiple hematopoietic lineages. Science. 1994;265:1573–7. 8079170 10.1126/science.8079170

[18] Sturrock A, Franklin KF, Hoidal JR. Human proteinase-3 expression is regulated by PU.1 in conjunction with a cytidine-rich element*. J Biol Chem. 1996;271:32392–402. 8943304 10.1074/jbc.271.50.32392

[19] Srikanth S, Rado TA. PU.1 regulates the expression of the human neutrophil elastase gene. Biochim Biophys Acta Gene Struct Expr. 1998;1398:215–23. 10.1016/s0167-4781(98)00039-6 9689920

[20] Karatepe K, Zhu H, Zhang X, Guo R, Kambara H, Loison F, et al. Proteinase 3 limits the number of hematopoietic stem and progenitor cells in murine bone marrow. Stem Cell Rep. 2018;11:1092–105. 10.1016/j.stemcr.2018.10.004 PMC6235012 30392974

[21] Lutz PG, Moog-Lutz C, Coumau-Gatbois E, Kobari L, Gioia YD, Cayre YE. Myeloblastin is a granulocyte colony-stimulating factor-responsive gene conferring factor-independent growth to hematopoietic cells. Proc Natl Acad Sci USA. 2000;97:1601–6. 10677505 10.1073/pnas.97.4.1601 PMC26481

[22] Garg B, Mehta HM, Wang B, Kamel R, Horwitz MS, Corey SJ. Inducible expression of a disease-associated ELANE mutation impairs granulocytic differentiation, without eliciting an unfolded protein response Mutant ELANE impairs differentiation. J Biol Chem. 2020;295:7492–7500. 32299910 10.1074/jbc.RA120.012366 PMC7247317

[23] Cheung P, Schaffert S, Chang SE, Dvorak M, Donato M, Macaubas C, et al. Repression of CTSG, ELANE and PRTN3-mediated histone H3 proteolytic cleavage promotes monocyte-to-macrophage differentiation. Nat Immunol. 2021;22:711–22. 34017121 10.1038/s41590-021-00928-y PMC8159908

[24] Zhu D, Hölz S, Metzger E, Pavlovic M, Jandausch A, Jilg C, et al. Lysine-specific demethylase 1 regulates differentiation onset and migration of trophoblast stem cells. Nat Commun. 2014;5:3174. 24448552 10.1038/ncomms4174

[25] Duteil D, Tosic M, Lausecker F, Nenseth HZ, Müller JM, Urban S, et al. Lsd1 ablation triggers metabolic reprogramming of brown adipose tissue. Cell Rep. 2016;17:1008–21. 27760309 10.1016/j.celrep.2016.09.053 PMC5081406

[26] Forneris F, Binda C, Dall’Aglio A, Fraaije MW, Battaglioli E, Mattevi A. A highly specific mechanism of histone H3-K4 recognition by histone demethylase LSD1*. J Biol Chem. 2006;281:35289–95. 16987819 10.1074/jbc.M607411200

[27] Walkley C, Yuan Y-D, Chandraratna R, McArthur G. Retinoic acid receptor antagonism in vivo expands the numbers of precursor cells during granulopoiesis. Leukemia. 2002;16:1763–72. 12200692 10.1038/sj.leu.2402625

[28] Hestdal K, Ruscetti FW, Ihle JN, Jacobsen SE, Dubois CM, Kopp WC, et al. Characterization and regulation of RB6-8C5 antigen expression on murine bone marrow cells. J Immunol. 1991;147:22–28. 1711076

[29] Dempster JM, Boyle I, Vazquez F, Root DE, Boehm JS, Hahn WC, et al. Chronos: a cell population dynamics model of CRISPR experiments that improves inference of gene fitness effects. Genome Biol. 2021;22:343. 34930405 10.1186/s13059-021-02540-7 PMC8686573

[30] Fishilevich S, Nudel R, Rappaport N, Hadar R, Plaschkes I, Stein TI, et al. GeneHancer: genome-wide integration of enhancers and target genes in GeneCards. Database. 2017;2017:bax028. 28605766 10.1093/database/bax028 PMC5467550

[31] Fiskus W, Mill CP, Nabet B, Perera D, Birdwell C, Manshouri T, et al. Superior efficacy of co-targeting GFI1/KDM1A and BRD4 against AML and post-MPN secondary AML cells. Blood Cancer J. 2021;11:98. 34016956 10.1038/s41408-021-00487-3 PMC8138012

[32] Vinyard ME, Su C, Siegenfeld AP, Waterbury AL, Freedy AM, Gosavi PM, et al. CRISPR-suppressor scanning reveals a nonenzymatic role of LSD1 in AML. Nat Chem Biol. 2019;15:529–39. 30992567 10.1038/s41589-019-0263-0 PMC7679026

[33] Maiques-Diaz A, Lynch JT, Spencer GJ, Somervaille TCP. LSD1 inhibitors disrupt the GFI1 transcription repressor complex. Mol Cell Oncol. 2018;5:e1481813. 30250927 10.1080/23723556.2018.1481813 PMC6150006

[34] Saleque S, Cameron S, Orkin SH. The zinc-finger proto-oncogene Gfi-1b is essential for development of the erythroid and megakaryocytic lineages. Gene Dev. 2002;16:301–6. 11825872 10.1101/gad.959102 PMC155332

[35] Kaufmann KB, Gründer A, Hadlich T, Wehrle J, Gothwal M, Bogeska R, et al. A novel murine model of myeloproliferative disorders generated by overexpression of the transcription factor NF-E2. J Exp Med. 2012;209:35–50. 22231305 10.1084/jem.20110540 PMC3260873

[36] Wehrle J, Seeger TS, Schwemmers S, Pfeifer D, Bulashevska A, Pahl HL. Transcription factor nuclear factor erythroid-2 mediates expression of the cytokine interleukin 8, a known predictor of inferior outcome in patients with myeloproliferative neoplasms. Haematologica. 2013;98:1073–80. 23445878 10.3324/haematol.2012.071183 PMC3696611

[37] Jutzi JS, Kleppe M, Dias J, Staehle HF, Shank K, Teruya-Feldstein J, et al. LSD1 inhibition prolongs survival in mouse models of MPN by selectively targeting the disease clone. Hemasphere. 2018;2:e54. 31723778 10.1097/HS9.0000000000000054 PMC6745991

[38] Duteil D, Metzger E, Willmann D, Karagianni P, Friedrichs N, Greschik H, et al. LSD1 promotes oxidative metabolism of white adipose tissue. Nat Commun. 2014;5:4093. 24912735 10.1038/ncomms5093 PMC4112219

[39] Jutzi JS, Basu T, Pellmann M, Kaiser S, Steinemann D, Sanders MA, et al. Altered NFE2 activity predisposes to leukemic transformation and myelosarcoma with AML-specific aberrations. Blood. 2019;133:1766–77. 30755419 10.1182/blood-2018-09-875047 PMC6484461

[40] Helness A, Fraszczak J, Joly-Beauparlant C, Bagci H, Trahan C, Arman K, et al. GFI1 tethers the NuRD complex to open and transcriptionally active chromatin in myeloid progenitors. Commun Biol. 2021;4:1356. 34857890 10.1038/s42003-021-02889-2 PMC8639993

[41] Arrigoni L, Al-Hasani H, Ramírez F, Panzeri I, Ryan DP, Santacruz D, et al. RELACS nuclei barcoding enables high-throughput ChIP-seq. Commun Biol. 2018;1:214. 30534606 10.1038/s42003-018-0219-z PMC6281648

[42] Langmead B, Salzberg SL. Fast gapped-read alignment with Bowtie 2. Nat Methods. 2012;9:357–9. 22388286 10.1038/nmeth.1923 PMC3322381

[43] Zhang Y, Liu T, Meyer CA, Eeckhoute J, Johnson DS, Bernstein BE, et al. Model-based analysis of ChIP-Seq (MACS). Genome Biol. 2008;9:R137. 18798982 10.1186/gb-2008-9-9-r137 PMC2592715

[44] Amemiya HM, Kundaje A, Boyle AP. The ENCODE blacklist: identification of problematic regions of the genome. Sci Rep. 2019;9:9354. 31249361 10.1038/s41598-019-45839-z PMC6597582

[45] Heinz S, Benner C, Spann N, Bertolino E, Lin YC, Laslo P, et al. Simple combinations of lineage-determining transcription factors prime cis-regulatory elements required for macrophage and B cell identities. Mol Cell. 2010;38:576–89. 20513432 10.1016/j.molcel.2010.05.004 PMC2898526

[46] Ramírez F, Ryan DP, Grüning B, Bhardwaj V, Kilpert F, Richter AS, et al. deepTools2: a next generation web server for deep-sequencing data analysis. Nucleic Acids Res. 2016;44:W160–W165. 27079975 10.1093/nar/gkw257 PMC4987876

[47] Community TG, Abueg LAL, Afgan E, Allart O, Awan AH, Bacon WA, et al. The Galaxy platform for accessible, reproducible, and collaborative data analyses: 2024 update. Nucleic Acids Res. 2024;52:W83–W94. 38769056 10.1093/nar/gkae410 PMC11223835

[48] Frankish A, Diekhans M, Ferreira A-M, Johnson R, Jungreis I, Loveland J, et al. GENCODE reference annotation for the human and mouse genomes. Nucleic Acids Res. 2019;47:D766–D773. 30357393 10.1093/nar/gky955 PMC6323946

[49] Patro R, Duggal G, Love MI, Irizarry RA, Kingsford C. Salmon provides fast and bias-aware quantification of transcript expression. Nat Methods. 2017;14:417–9. 28263959 10.1038/nmeth.4197 PMC5600148

[50] Soneson C, Love MI, Robinson MD. Differential analyses for RNA-seq: transcript-level estimates improve gene-level inferences. F1000Research. 2016;4:1521. 10.12688/f1000research.7563.1 PMC4712774 26925227

[51] Love MI, Huber W, Anders S. Moderated estimation of fold change and dispersion for RNA-seq data with DESeq2. Genome Biol. 2014;15:550. 25516281 10.1186/s13059-014-0550-8 PMC4302049

[52] Young MD, Behjati S. SoupX removes ambient RNA contamination from droplet-based single-cell RNA sequencing data. GigaScience. 2020;9:giaa151. 33367645 10.1093/gigascience/giaa151 PMC7763177

[53] Germain P-L, Lun A, Macnair MeixideCG, Robinson W. MD. doublet identification in single-cell sequencing data using scDblFinder. F1000Research. 2021;10:979. 35814628 10.12688/f1000research.73600.1 PMC9204188

[54] Wolf FA, Angerer P, Theis FJ. SCANPY: large-scale single-cell gene expression data analysis. Genome Biol. 2018;19:15. 29409532 10.1186/s13059-017-1382-0 PMC5802054

[55] Hao Y, Stuart T, Kowalski MH, Choudhary S, Hoffman P, Hartman A, et al. Dictionary learning for integrative, multimodal and scalable single-cell analysis. Nat Biotechnol. 2024;42:293–304. 37231261 10.1038/s41587-023-01767-y PMC10928517

[56] Tirosh I, Izar B, Prakadan SM, Wadsworth MH II, Treacy D, Trombetta JJ, et al. Dissecting the multicellular ecosystem of metastatic melanoma by single-cell RNA-seq. Science. 2016;352:189–96. 27124452 10.1126/science.aad0501 PMC4944528

[57] Korsunsky I, Millard N, Fan J, Slowikowski K, Zhang F, Wei K, et al. Fast, sensitive, and accurate integration of single cell data with Harmony. Nat methods. 2019;16:1289–96. 31740819 10.1038/s41592-019-0619-0 PMC6884693

[58] Gayoso A, Lopez R, Xing G, Boyeau P, Amiri VVP, Hong J, et al. A Python library for probabilistic analysis of single-cell omics data. Nat Biotechnol. 2022;40:163–6. 35132262 10.1038/s41587-021-01206-w

[59] Lopez R, Regier J, Cole MB, Jordan MI, Yosef N. Deep generative modeling for single-cell transcriptomics. Nat methods. 2018;15:1053–8. 30504886 10.1038/s41592-018-0229-2 PMC6289068

[60] Xu C, Lopez R, Mehlman E, Regier J, Jordan MI, Yosef N. Probabilistic harmonization and annotation of single-cell transcriptomics data with deep generative models. Mol Syst Biol. 2021;17:MSB20209620. 10.15252/msb.20209620 PMC7829634 33491336

[61] Chen Y, Chen L, Lun ATL, Baldoni PL, Smyth GK. edgeR v4: powerful differential analysis of sequencing data with expanded functionality and improved support for small counts and larger datasets. Nucleic Acids Res. 2025;53:gkaf018. 39844453 10.1093/nar/gkaf018 PMC11754124

