# Supplementary material for: Lysine-specific demethylase 1 regulates hematopoietic stem cell expansion and myeloid cell differentiation
Source: Cell Death Dis. 2025 Aug 15;16(1):619. doi: 10.1038/s41419-025-07951-z (PMC12354751; doi:10.1038/s41419-025-07951-z)
Supplement: Supplementary file 1 — Supplemental Figures [file 41419_2025_7951_MOESM1_ESM.docx]

**
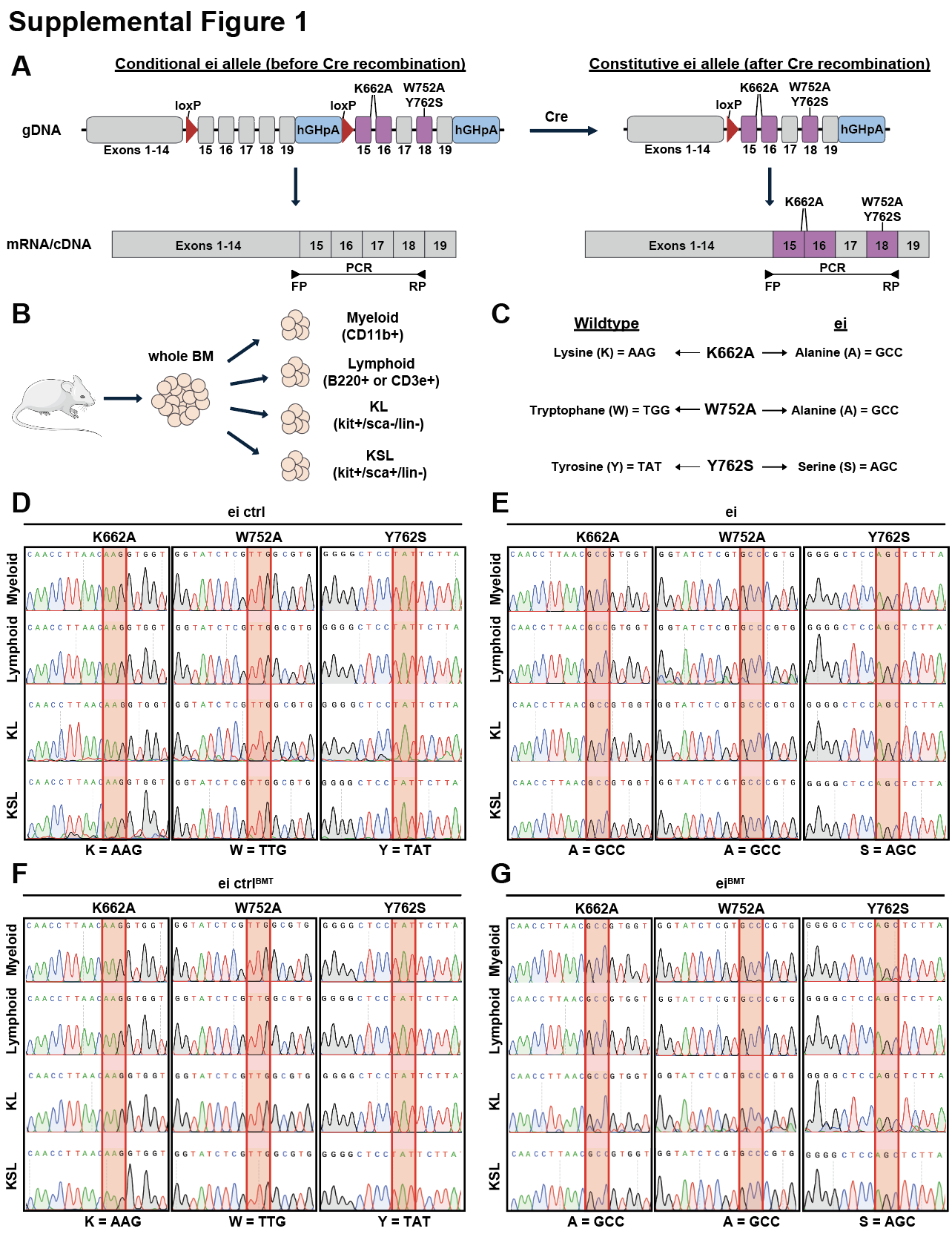
**

**Supplemental Figure 1**: **Validation of Tamoxifen-induced recombination in the *Lsd1* ei mouse model.** (**A**) Schematic representation of the *Lsd1* allele (top) and the resulting mRNA (bottom) before (left) and after (right) Cre recombination. (**B**) Experimental procedure: whole bone marrow (BM) was sorted to obtain myeloid (CD11b^+^), lymphoid (B220^+^ or CD3e^+^), KL (kit^+^/sca^-^/lin^-^) and KSL (kit^+^/sca^+^/lin^-^) cells for RNA isolation and reverse transcription. Subsequently, the region of interest was amplified by PCR to interrogate the cDNA for the three ei mutations (K662A, W752A and Y762S). (**C**) DNA sequences and resulting amino acid translation in wt control and mutated ei animals. (**D-G**) Sanger sequencing of (**D**) *Lsd1* ei ctrl, (**E**) *Lsd1* ei, (**F**) *Lsd1* ei ctrl^BMT^ and (**G**) *Lsd1* ei^BMT^ mice. The amino acids targeted for mutation are highlighted in red.

**
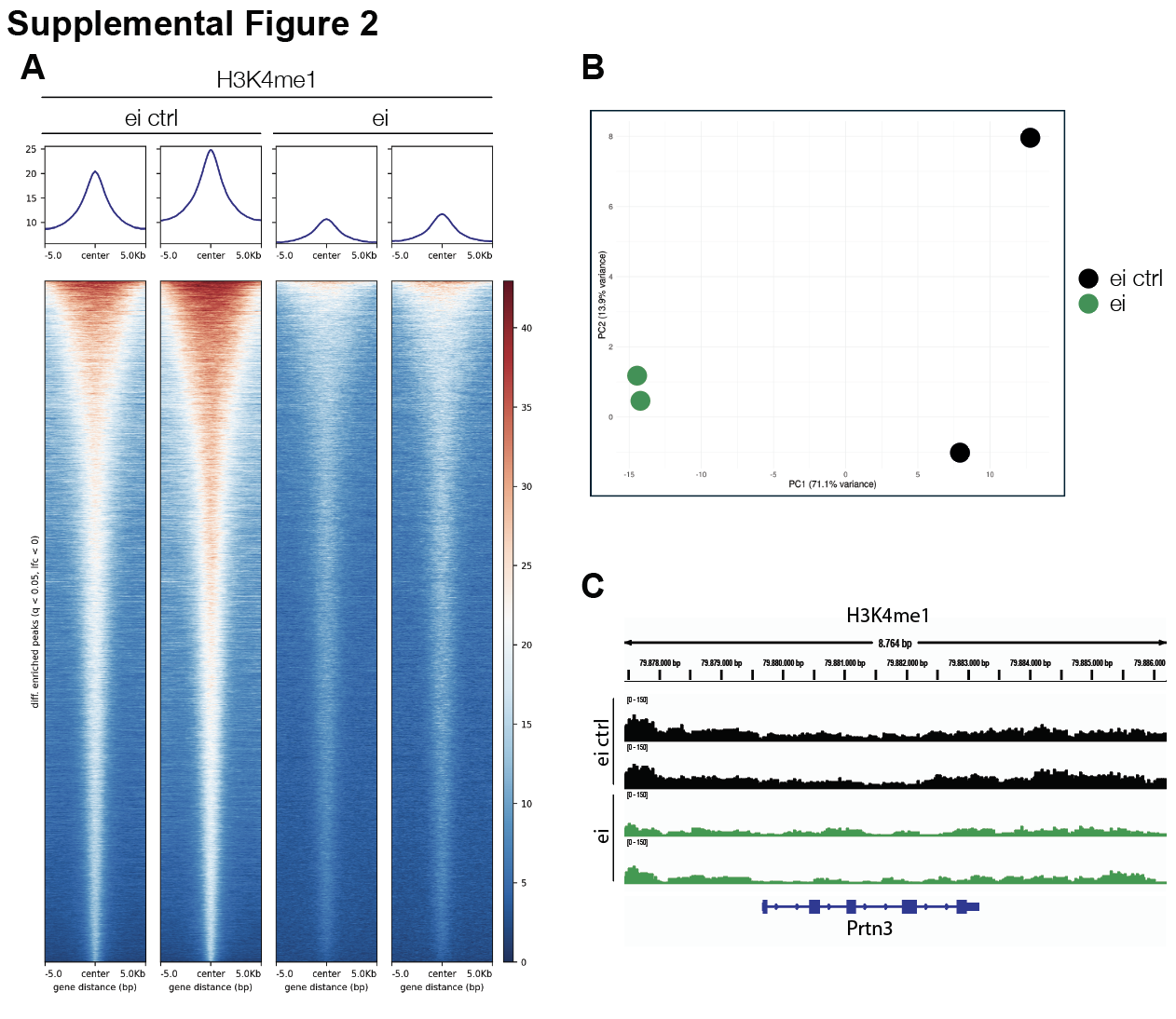
**

**Supplemental Figure 2: Loss of Lsd1 enzymatic function leads to global changes in H3K4me1 levels**. (**A**) Heat map of H3K4me1 ChIP-seq peaks in CD117-positive cells from *Lsd1* ei ctrl and *Lsd1* ei mice, n=2 independent mice of each genotype. (**B**) PCA plot of the samples shown in (**A**). (**C**) Integrative Genomics Viewer (IGV) tracks of H3K4me1 ChIP-seq at the *Prtn3* locus. *Lsd1* ei ctr mice are shown in black and *Lsd1* ei mice in green.

**
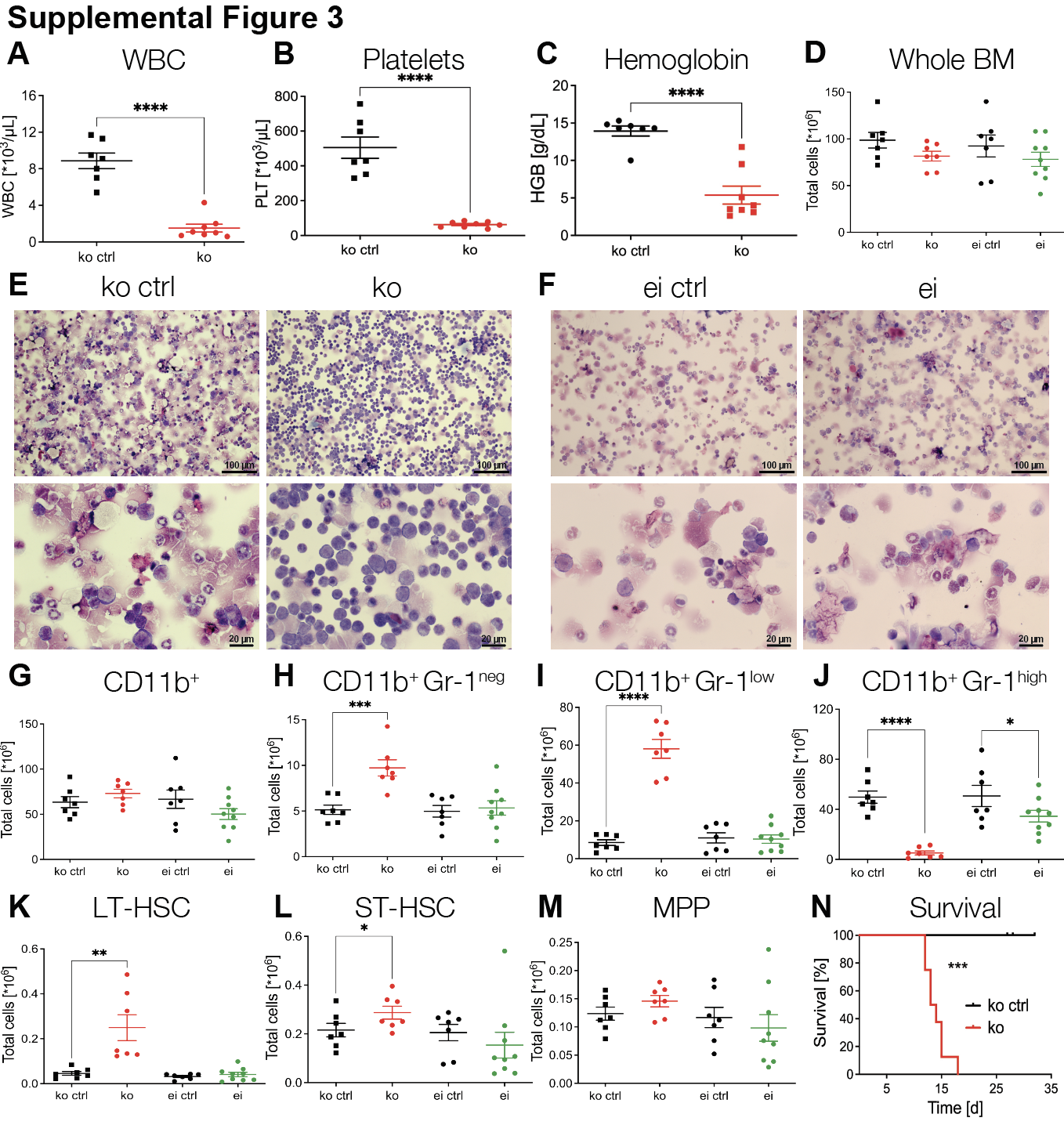
**

**Supplemental Figure 3: *Lsd1* knockout mice display pancytopenia in the peripheral blood, altered bone marrow morphology and stem and progenitor cell composition, as well as decreased survival.** (**A-N**) Phenotype of *Lsd1* ko^BMT^ and Lsd1 ei^BMT^ mice, *Lsd1^fl/fl^* mice without Cre served as controls. (**A-D, G-M**) n=7-8 per genotype. Statistical analysis was performed using Student’s t tests. *p<0.05; **p<0.01; ***p<0.001; ****p<0.0001. (**A**) White blood cell counts (WBC), (**B**) Platelet (PLT) counts, and (**C**) hemoglobin (HGB) values. (**D**) Absolute cell counts of whole BM isolated from one femur, two tibiae, pelvic bones, the sternum and the spine. (**E+F**) May-Grünwald Giemsa (MGG) stained BM cytospins: (**E**) *Lsd1* ko and *Lsd1* ko ctrl (**F**) *Lsd1* ei mice and *Lsd1* ei ctrl. 200x magnifications (top) and 630x magnifications (bottom). (**G-J**) Absolute number of myeloid cells in the BM of *Lsd1* ko and *Lsd1* ei mice: (**G**) CD11b^+^ positive cells, (**H**) CD11b^+^ Gr-1^neg^, (**I**) CD11b^+^ Gr-1^low^, (**J**) CD11b^+^ Gr-1^high^. (**K-M**) Absolute number of hematopoietic stem and progenitor cells in the BM of *Lsd1* ko and *Lsd1* ei mice: (**K**) long-term HSCs (LT-HSC), (**L**) short-term HSCs (ST-HSC), and (**M**) multipotent progenitors (MPP). (**N**) Kaplan-Meier survival curves of *Lsd1* ko and control mice, performed using Log-Rank (Mantel-Cox) testing. ***p<0.001.

**
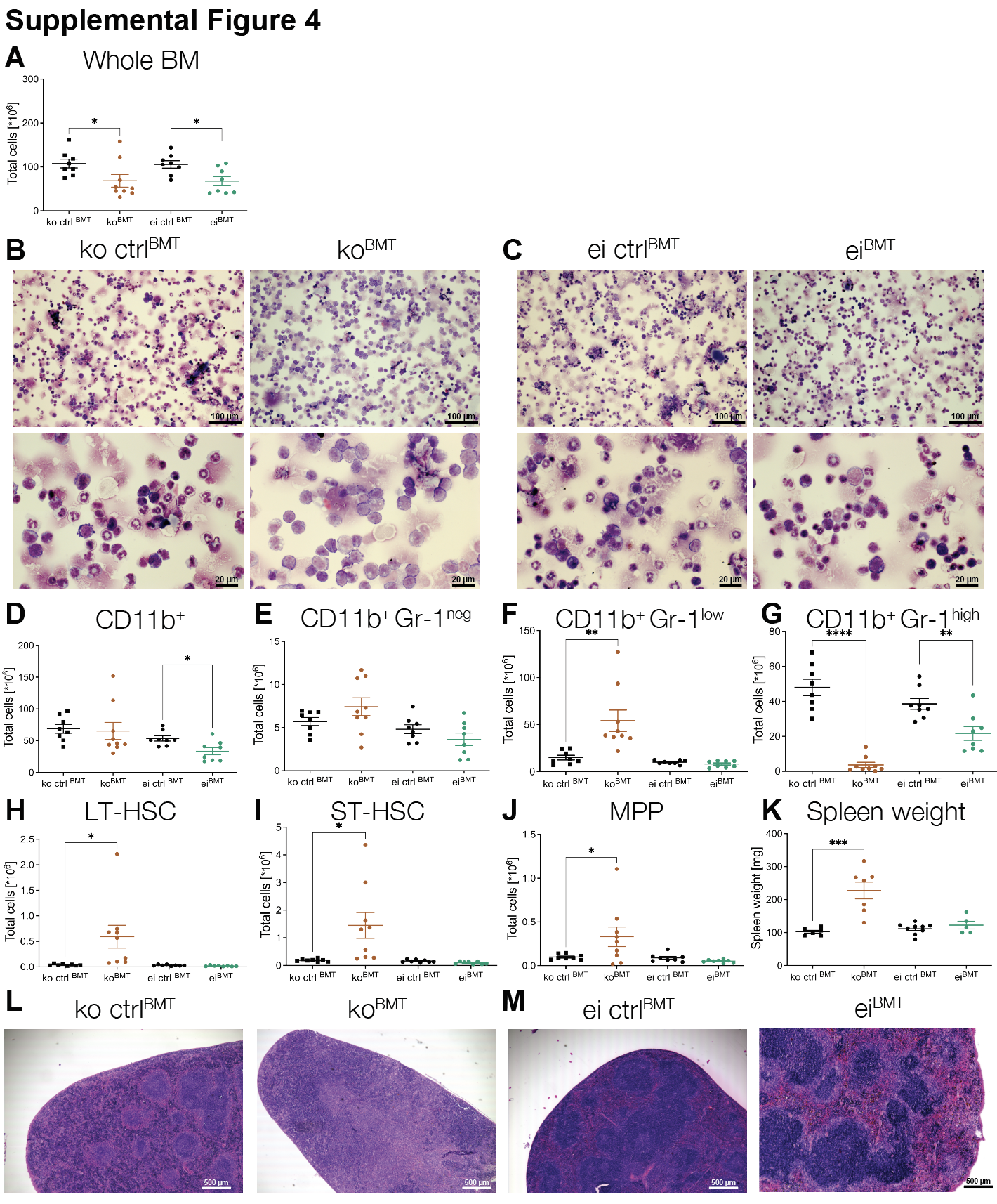
**

**Supplemental Figure 4: Additional phenotypic characterization of *Lsd1* ko^BMT^ and *Lsd1* ei^BMT^ mice.** (**A-M**) Phenotype of *Lsd1* ko^BMT^ and *Lsd1* ei^BMT^ mice, *Lsd1*^fl/fl^ mice without Cre served as separate controls for both mouse lines (*Lsd1* ko ctr^BMT^ and *Lsd1* ei ctr^BMT^). (**A, D-K**) n=5-9 per genotype. Statistical testing on blood values and BM was performed using Student’s t tests. *p<0.05; **p<0.01; ****p<0.0001. (**A**) Absolute cell counts of whole BM per mouse: cells isolated from one femur, two tibiae, pelvic bones, the sternum and the spine were enumerated. (**B+C**) May-Grünwald Giemsa (MGG) stained cytospins of BM from (**B**) *Lsd1* ko^BMT^ and *Lsd1* ko ctrl^BMT^ mice, (**C**) *Lsd1* ei^BMT^ and *Lsd1* ei ctrl^BMT^ mice. 200x magnifications (top) and 630x magnifications (bottom). (**D-J**) Absolute cell counts of myeloid as well as hematopoietic stem and progenitor (HSPC) cells in the BM of *Lsd1* ko^BMT^, *Lsd1* ei^BMT^ mice and their respective controls: (**D**) CD11b^+^ positive cells, (**E**) CD11b^+^ Gr-1^neg^, (**F**) CD11b^+^ Gr-1^low^, (**G**) CD11b^+^ Gr-1^high^, (**H**) long-term HSCs (LT-HSC), (**I**) short-term HSCs (ST-HSC), and (**J**) multipotent progenitors (MPP). (**K**) Spleen weights of *Lsd1* ko^BMT^, *Lsd1* ei^BMT^, and control mice. (**L+M**) Histopathological slides of (**L**) *Lsd1* ko ctrl^BMT^ (left) and *Lsd1* ko^BMT^ (right) as well as (**M**) *Lsd1* ei ctrl^BMT^ (left) and *Lsd1* ei^BMT^ (right). 40x magnifications.

**Supplemental Figure 5: Reference cell type labeling of control mice and validation of canonical cell type markers.** (**A**) Heatmap of expression levels of the top 10 marker genes for each reference cell type ordered by log-fold-change and filtered for at least 25% expression in the relevant population. (**B**) UMAP embedding obtained from harmonized principal component analyses across n=7 control mice (*Lsd1* ei ctrl, *Lsd1* ko ctrl, and *Lsd1* ko ctrl^BMT^ combined). Cells are colored by reference cell type. A small cluster of cells that passed quality control, but had no clear marker genes, termed ”mixed undetermined” was excluded from further analyses. (**C-Q**) UMAP plots of harmonized control cells, colored by expression of the following markers genes: (**C**) lactotransferrin, *Ltf.* (**D**) CCAAT/enhancer binding protein epsilon, *Cebpe.* (**E**) coagulation factor XIII, A1 subunit, *F13a1.* (**F**) PCNA clamp associated factor (G1/S transition: marks cycling cells / promonocytes), *Pclaf.* (**G**) aminolevulinic acid synthase, *Alas2.* (**H**) T-cell surface glycoprotein CD3 epsilon chain, *Cd3*ε. (**I**) Proteinase 3, *Prtn3.* (**J**) immunoglobulin joining chain, *Jchain.* (**K**) sialic acid binding Ig-like lectin, *Siglech.* (**L**) histocompatibility 2, class II antigen E beta, *H2-Eb1.* (**M**) paired box 5, *Pax5.* (**N**) adhesion G protein-coupled receptor E4, *Adgre4.* (**O**) carboxypeptidase A3, *Cpa3.* (**P**) chemokine (C motif) receptor 1, *Xcr1.* (**Q**) MDS1 and EVI1 complex locus, *Mecom*.

**Supplemental Figure 6: Expression of hematopoietic stem and progenitor (HSPC) cell markers in *Lsd1* control and Lsd1 ko/ko^BMT^ mice.** (**A-E**) UMAP embeddings obtained from harmonized principal component analyses across n=7 control mice (*Lsd1* ei ctrl, *Lsd1* ko ctrl and *Lsd1* ko ctrl^BMT^ combined), colored by: (**A**) manually assigned reference cell type, (**B-E**) expression of the following marker genes: (**B**) *Mecom* (MDS1 and EVI1 complex locus), (**C**) *Cd34* (CD34 antigen), (**D**) *Hlf* (hepatic leukemia factor), and (**E**) *Meis1* (Meis homeobox 1). (**F-J**) UMAP embeddings obtained from harmonized principal component analyses across n=2 *Lsd1* ko and n=2 *Lsd1* ko^BMT^ mice, colored by: (**F**) cell type predicted by scANVI, (**G-J**) expression of the following marker genes: (**G**) *Mecom,* (**H**) *Cd34*, (**I**) *Hlf*, and (**J**) *Meis1*.

**Supplemental Figure 7: Loss of LSD1 protein decreases lymphoid output.** (**A-D**) Lymphoid cells in the BM of *Lsd1* ko^BMT^ and *Lsd1* ei^BMT^ mice as well as their respective controls. Statistical analyses were conducted using Student’s t tests. ***p<0.001; ****p<0.0001. n=7-9 per genotype. (**A+B**) B-cells (B220^+^) and (**C+D**) T-cells (CD3ε^+^) are shown as (**A+C**) frequency by flow cytometry analysis and (**B+D**) absolute cell numbers.

**Supplemental Figure 8: LSD1 regulates GFI1 binding.** (**A**) Integrative Genomics Viewer (IGV) tracks of LSD1 and SPI1 ChIP-seqs in murine primary cells. The *PRTN3* locus is shown. The LSD1-ChIP was performed by Whyte *et al.* on murine embryonic stem cells (ESC). [1] The SPI1-ChIP was performed by Chavez *et al.* on murine ESC. [2] (**B**) Known LSD1, SPI1, and GFI1 binding sites. IGV tracks of the LSD1 (red), the SPI1 (green) and the GFI1 (blue) ChIP-seqs. Input controls (black). The *ITGAM* (up), *GFI1* (middle) and *CD86* (bottom) loci are shown. The SPI1 and GFI1 ChIP-seqs were performed in THP1 cells. LSD1 ChIP-seq data set by Ravasio *et. al.* on NB4 cells. [3] (**C**) HOMER motif analysis of the LSD1, SPI1, and GFI1 ChIP-seq experiments in NB4 and THP1 cells. (**D**) Heat maps of the 498 GFI1 peaks identified in non-targeted K562 cells (sg-NT) (see Fig. 4G): Depicted is LSD1 and GFI1 binding at these sites in K562 sg-NT cells as well as GFI1 binding at these sites in LSD1 depleted (sg-LSD1) K562 cells. (**E**) Heat maps of the 6,548 LSD1 peaks identified in non-targeted K562 cells (sg-NT) (see Fig. 4G): Depicted is LSD1 and GFI1 binding at these sites in K562 sg-NT cells as well as GFI1 binding at these sites in LSD1 depleted (sg-LSD1) K562 cells.

**Supplemental Figure 9: Phenotypic effects of *Prtn3* knockdown *in vivo*.** (**A-K**) kit^+^ BM cells either from wt animals (**A**) or from *Lsd1* ko animals (**B-K**) were transduced with either a scrambled-shRNA control virus (scr) or the shRNA1 against *Prtn3* (see Fig. 5A, *Prtn3* kd) and transplanted as shown in Fig. 5E. (**A+B**) *Prtn3* RNA expression measured by qRT-PCR in (**A**) wt BM cells three days after transduction with shRNA 1 and in (**B**) *Lsd1* ko BM, harvested three weeks after transplantation. (**C-K**) Phenotype of *Lsd1* ko BM transduced either with scrambled-shRNA control virus (scr) or an shRNA against *Prtn3* (*Prtn3* kd). (**C-E** and **H-J**) n=4-5 per genotype. Statistical analysis was conducted using Student’s t tests. *p<0.05. (**C-E**) Complete blood counts: (**C**) White blood cell counts (WBC), (**D**) Platelet (PLT) counts, and (**E**) hemoglobin (HGB). (**F+G**) May-Grünwald Giemsa (MGG) stained blood smears. 1000x magnifications. (**H-J**) Differential blood counts: (**H**) lymphocytes, (**I**) granulocytes, and (**J**) monocytes. (**K**) MGG-stained BM cytospins. 200x magnifications (top) and 630x magnifications (bottom).

**Supplemental Figure 10: Phenotypic characterization of colonies generated by wt, *Lsd1* ko and *Lsd1* ko *Prtn3*-depleted BM.** (**A-H**) Colony assays of kit^+^ wild type (**A, B, E, F**) and *Lsd1* ko (**C, D, G, H**) bone marrow transduced either with a control scrambled shRNA (scr) (**A, C, E, G**) or with shRNA1 against *Prtn3* (*Prtn3* kd, see Fig. 5A) (**B, D, F, H**). (**A-D**) Light microscopy photographs of colony assays: overview (top line) and single colonies (lines below). The colony type assigned for enumeration is shown in the top left corner. (**E-H**) May-Grünwald Giemsa (MGG) stained cytospins of single colonies. 630x magnifications. The colony type assigned for enumeration is shown in the top left corner. CFU (colony-forming unit). GEMM (granulocyte-erythrocyte-monocyte-megakaryocyte). E (erythrocyte). GM (granulocyte-monocyte). G (granulocyte). M (monocyte). GM/G/M (aberrant granulocytic and monocytic appearing colonies in *Lsd1* ko mice).

**References**

1 Whyte WA, Bilodeau S, Orlando DA, Hoke HA, Frampton GM, Foster CT *et al.* Enhancer decommissioning by LSD1 during embryonic stem cell differentiation. *Nature* 2012; **482**: 221–225.

2 Chavez JS, Rabe JL, Loeffler D, Higa KC, Hernandez G, Mills TS *et al.* PU.1 enforces quiescence and limits hematopoietic stem cell expansion during inflammatory stress. *J Exp Med* 2021; **218**: e20201169.

3 Ravasio R, Ceccacci E, Nicosia L, Hosseini A, Rossi PL, Barozzi I *et al.* Targeting the scaffolding role of LSD1 (KDM1A) poises acute myeloid leukemia cells for retinoic acid–induced differentiation. *Sci Adv* 2020; **6**: eaax2746.
