# Supplementary figures and images for: Lysine-specific demethylase 1 regulates hematopoietic stem cell expansion and myeloid cell differentiation
Source: Cell Death Dis. 2025 Aug 15;16(1):619. doi: 10.1038/s41419-025-07951-z (PMC12354751; doi:10.1038/s41419-025-07951-z)

Supplemental Figure 11

A

shown in Fig. 4F

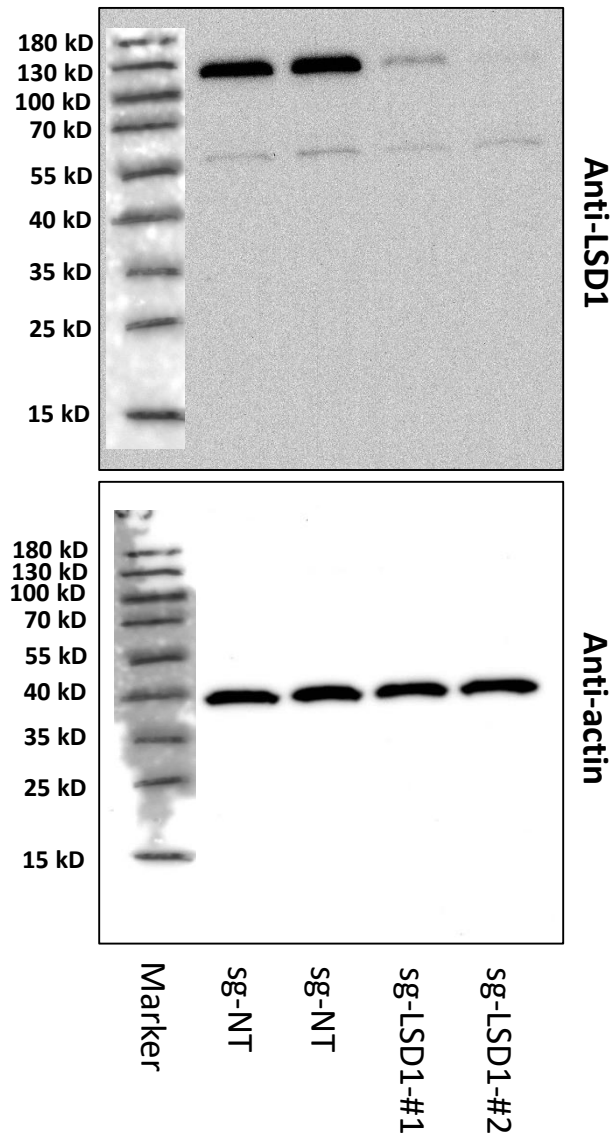

B

shown in Fig. 5A

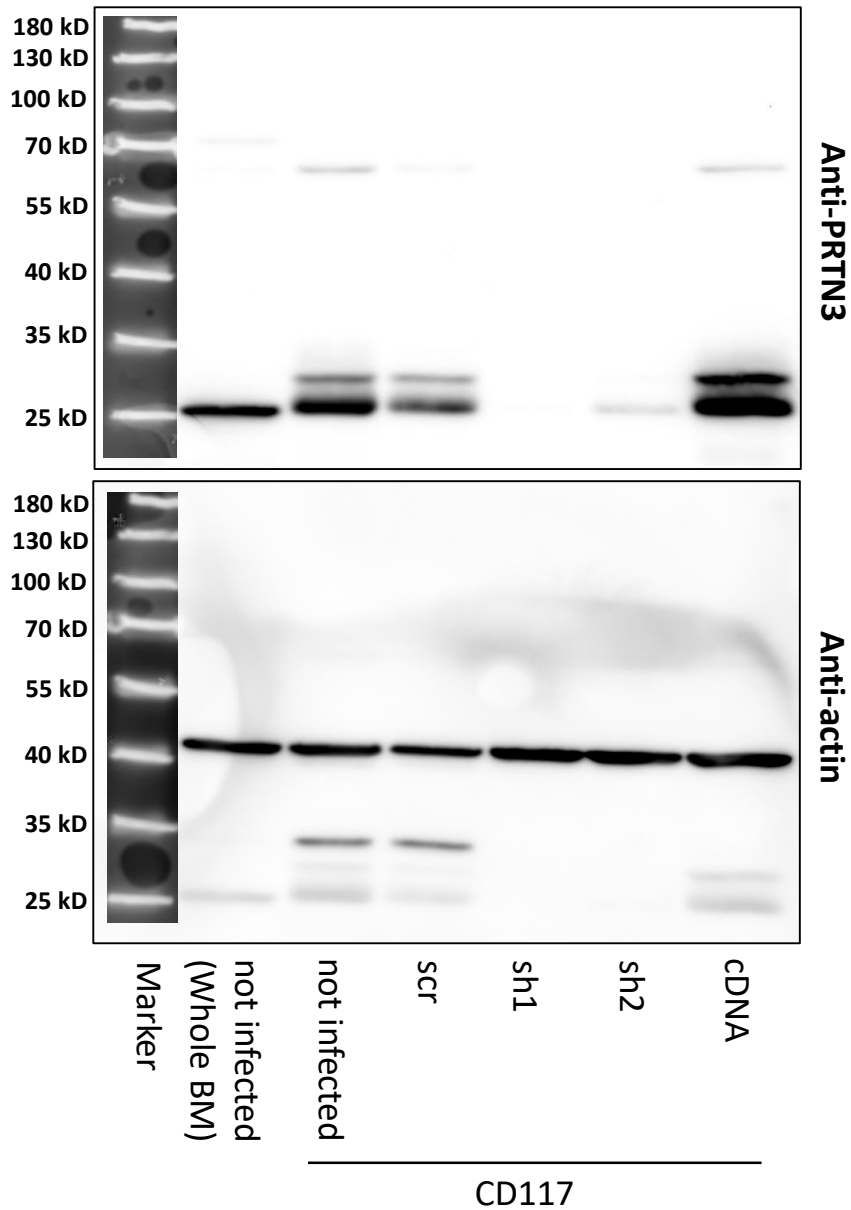

Supplement: Supplementary file 3 — Uncropped Western Blots [file 41419_2025_7951_MOESM3_ESM.pdf]
